# Force‐Electric Nanotentacles of Tantalum Implant Accelerate Cellular Energy Metabolism/Crosstalk for Immunomodulation‐Vascularized Bone Integration

**DOI:** 10.1002/advs.77514

**Published:** 2026-08-30

**Authors:** Taixing Zhang, Ke Ma, Kangqing Zuo, Pandong Lin, Tailong Zhang, Rongliang Ding, Linbo Zhang, Aonan Li, Yinchuan Wang, Yanling Huang, Jichao Feng, Guiyong Xiao, Yupeng Lu, Bing Han, Zhiqiang Wang, Ningbo Li

**Affiliations:** ^1^ Department of Stomatology Shandong Provincial Hospital Affiliated to Shandong First Medical University Ji'nan Shandong Province P. R. China; ^2^ School of Stomatology Shandong First Medical University & Shandong Academy of Medical Sciences Ji'nan Shandong Province P. R. China; ^3^ Medical Science and Technology Innovation Center Shandong First Medical University & Shandong Academy of Medical Sciences Ji'nan Shandong Province P. R. China; ^4^ Key Laboratory for Liquid‐Solid Structural Evolution and Processing of Materials Ministry of Education Shandong University Ji'nan P. R. China; ^5^ School of Materials Science and Engineering Shandong University Ji'nan P. R. China; ^6^ Department of Orthodontics Shandong Provincial Hospital Affiliated to Shandong First Medical University Ji'nan Shandong Province P. R. China; ^7^ School of Radiology Shandong First Medical University & Shandong Academy of Medical Sciences Taian Shandong Province P. R. China; ^8^ Department of Orthodontics, Cranial‐Facial Growth and Development Center Peking University School and Hospital of Stomatology & National Center of Stomatology & National Clinical Research Center for Oral Diseases & National Engineering Laboratory for Digital and Material Technology of Stomatology & Beijing Key Laboratory for Digital Stomatology & Research Center of Engineering and Technology for Computerized Dentistry Ministry of Health & NMPA Key Laboratory for Dental Materials Haidian District Beijing P.R. China

**Keywords:** force‐electrical nanotentacles, immune‐osteogenic crosstalk, immunomodulation, mitochondrial, tantalum implant, vascularized bone integration

## Abstract

Current load‐bearing bone implants primarily serve as static structural supports, still fall short of replicating the dynamic, spatiotemporal, and multifunctional coordination inherent in natural bone regeneration. Herein, we first develop a biomimetic force‐electric responsive tantalum (Ta) implant with surface‐nanostructured LiTaO_3_ (n‐ LiTaO_3_) via combining laser nanofabrication and Li^+^‐induced in situ reaction, achieving favorable immunomodulation‐mediated vascularized osseointegration under low‐intensity pulsed ultrasound stimulation. The piezoelectric LiTaO_3_ nanotentacles convert ultrasound vibration into localized electrical signals, triggering Ca^2^^+^ influx, accelerating mitochondrial ATP production, and activating PI3K–AKT signaling pathway in BMSCs to promote osteogenesis. It also orchestrates a pro‐osteoregenerative immune microenvironment through M2 macrophage polarization and immune–osteogenic crosstalk mediated by ECM–integrin–FAK axis. The force‐electric response implant yet promotes angiogenesis mainly via enhancing intracellular Ca^2^^+^‐dependent eNOS/NO cue and cellular metabolism, reduces the inflammatory response in the dorsal subcutaneous tissue of rats, and further promotes vascularization and bone integration in femoral defects. The novel force‐electric response Ta implant endows real‐time controllable regulation of multifunctional tissue regeneration for using as patient‐personalized load‐bearing osteoarticular prostheses.

## Graphical abstract

A biomimetic force‐electric nanotentacle‐enabled tantalum (Ta) implant with surface‐nanostructured LiTaO_3_ (n‐LiTaO_3_) was first developed via laser nanofabrication combined with Li^+^‐induced in situ reaction, enabling the transduction of ultrasound into bioelectrical cues. These electrical signals potentiate mitochondrial energetics and orchestrate a coordinated immunomodulatory (PI3K–AKT), angiogenic (eNOS/NO), and osteogenic (PI3K–AKT) network, while establishing a pro‐osteoregenerative immune microenvironment via M2 macrophage polarization and ECM–integrin–FAK‐mediated crosstalk, thereby driving osteoimmunomodulation‐coupled vascularized bone integration.

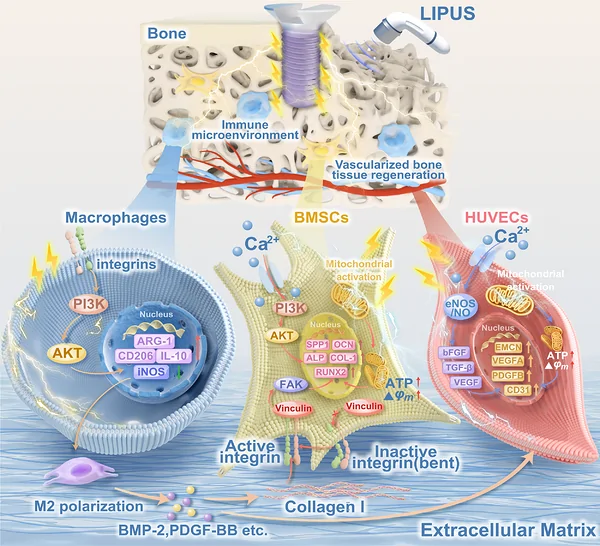

## 1 Introduction

The persistent failure of load‐bearing bone implants underscores a fundamental limitation of conventional biomaterials: their mono‐functional design fails to recapitulate the dynamic, multifunctional crosstalk, among mechanical, angiogenic, and immunomodulatory cues, that underpins native bone homeostasis and regeneration [1]. At the clinical level, stable and durable osseointegration represents the central biological endpoint of implant therapy, as it underpins long‐term load transfer, mechanical stability, and functional rehabilitation [2]. However, persistent challenges, including large segmental defects, compromised osseointegration in osteoporotic bone, and high failure rates in revision surgeries, largely stem from the disruption of the tightly coordinated biological processes required for effective bone regeneration [3, 4, 5]. Native bone healing exemplifies a highly orchestrated cascade in which immune regulation, angiogenesis, and osteogenesis are spatiotemporally integrated within a dynamic, multi‐scale network [6]. Although conventional metallic implants provide excellent mechanical support, their biological passivity often results in delayed osseointegration, insufficient vascularization, and persistent inflammation at the bone–implant interface [7, 8]. However, strategies such as bioactive coatings, growth factor delivery, and surface nanoengineering have been explored [9, 10], most remain passive or target isolated pathways, limiting their ability to coordinate the complex cellular crosstalk required for bone regeneration. These challenges highlight the need for a new generation of intelligent implants capable of actively programming the peri‐implant microenvironment [11, 12].

Vascularization is essential for bone regeneration by supplying oxygen and nutrients and coordinating osteogenesis, yet the peri‐implant region is often characterized by limited vascular supply and restricted mass transport [13]. Insufficient early vascularization leading to delayed healing, fibrous encapsulation, or implant failure [14]. So implant materials capable of actively promoting angiogenesis are essential for establishing a functional bone–implant interface. Piezoelectric materials incorporating BaTiO_3_ or polyvinylidene fluoride (PVDF) have been shown to enhance endothelial migration, tube formation, and VEGF secretion by generating localized electrical signals under mechanical or ultrasonic stimulation [15, 16, 17]. Given that endothelial activation, nitric oxide (NO) production, and vascular remodeling are tightly regulated by calcium‐dependent electrical signaling, piezoelectric surface are uniquely positioned to address the vascular bottleneck at the bone implant interface [16, 18]. In addition to promoting angiogenesis, immune regulation has become a core upstream determinant of peri‐implant angiogenesis and subsequent osteogenesis. In the implant setting, macrophage polarization governs inflammation resolution and critically dictates vascular ingrowth. M2‐polarized macrophages actively promote angiogenesis through the secretion of VEGF, PDGF‐BB, and TGF‐β, while simultaneously supporting osteogenic differentiation, thereby acting as a biological bridge between vascularization and bone formation [19]. Emerging evidence further indicates that bioelectric cues can directly influence macrophage polarization by modulating integrin‐associated signaling, highlighting immune cells as electrosensitive regulators rather than passive responders [20].

Among emerging strategies to restore such coordination, bioelectric signaling has attracted increasing attention as a fundamental yet underexploited regulator of bone regeneration. In native bone, the piezoelectric nature of collagen fibrils generates endogenous electrical potentials in response to mechanical loading, which serve as direct instructive cues for osteoblast activity, matrix mineralization, and anabolic remodeling [21]. These endogenous electric fields have been recognized as important regulators of osteogenesis, angiogenesis, and immune responses, coordinating multiple cellular processes involved in bone regeneration [22]. So translating this bioelectric principle into implant design highlights the importance of endowing implants with controllable electrical activity to generate localized, on‐demand bioelectric cues for regulating osteogenic regeneration. Piezoelectric biopolymers and bioceramics generate surface charges upon mechanical deformation due to their non‐centrosymmetric crystal structures, producing transient electric potentials that interact with ion channels, integrin‐mediated adhesion complexes, and intracellular signaling pathways [23, 24, 25]. Extensive efforts have focused on piezoelectric biomaterials such as PVDF, PLLA, BaTiO_3_, and KNN, which have demonstrated the ability to promote osteogenic differentiation, while also influencing angiogenic and immune responses through mechanoelectrical stimulation [26, 27]. Despite these encouraging biological effects, the translation of piezoelectric biomaterials to load‐bearing orthopedic applications remains limited. Polymeric piezoelectrics often lack sufficient mechanical strength and long‐term stability, while ceramic‐based systems, despite high piezoelectric coefficients, face challenges related to brittleness, interfacial mismatch, and processing incompatibility with metallic implants [28, 29].

**FIGURE 1:**
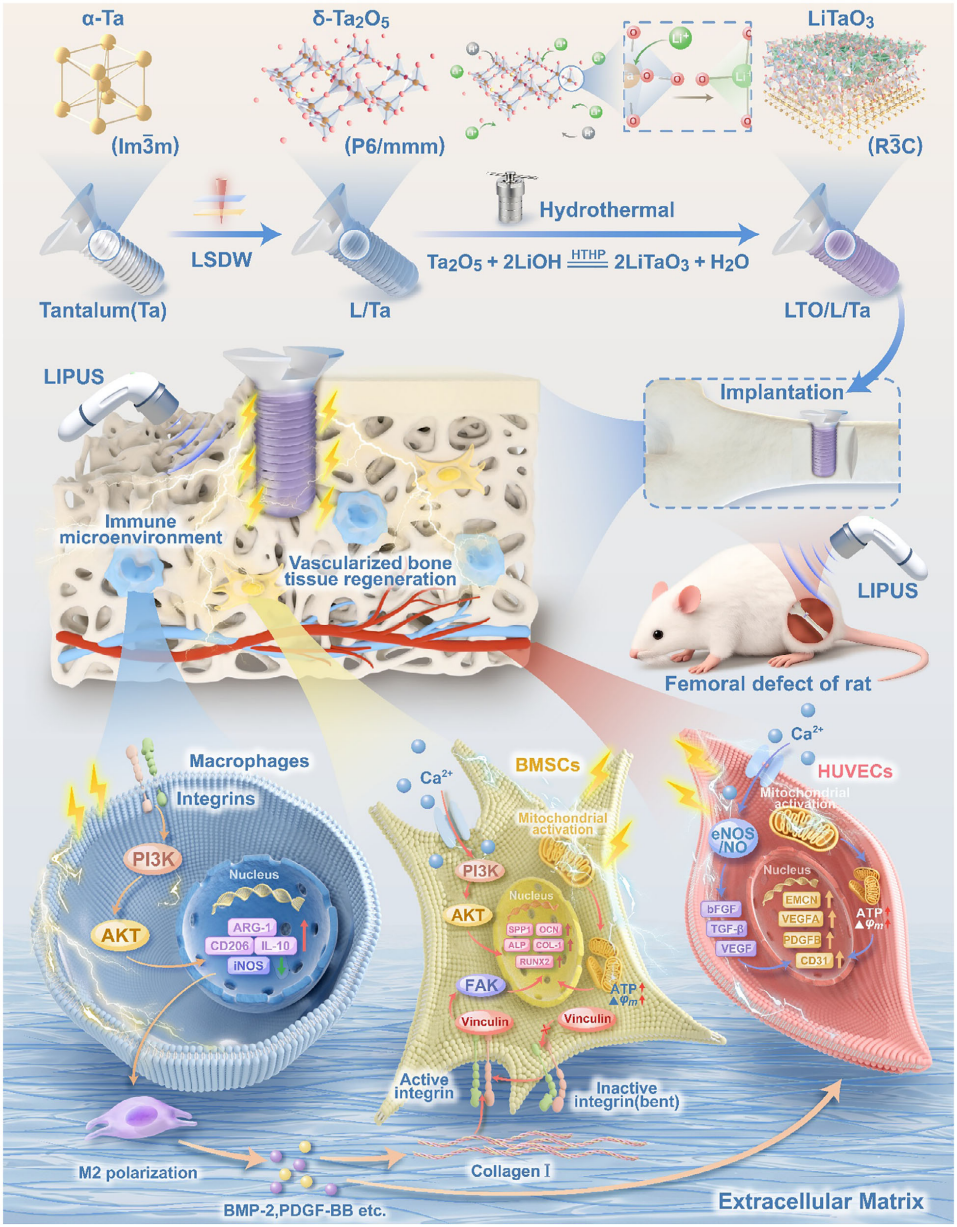
Schematic illustration of the fabrication, piezoelectric activation, and multilevel osteo‐angiogenic immunomodulatory mechanism of the force‐electric Ta implant.

Tantalum (Ta) has attracted increasing attention as an advanced implant material due to its excellent biocompatibility, outstanding corrosion resistance, and favorable osteointegration capability [30]. Beyond these intrinsic properties, tantalum has demonstrated unique clinical value in challenging load‐bearing orthopedic applications, including acetabular reconstruction during revision total hip arthroplasty and the treatment of large bone defects, owing to its excellent mechanical reliability and capacity for bone ingrowth [31]. These advantages make tantalum particularly attractive for next‐generation implants that require both long‐term structural stability and active biological regulation. Nevertheless, current tantalum implants primarily rely on their favorable structural properties for mechanical fixation and lack intrinsic bioactive functions capable of actively regulating the peri‐implant microenvironment. Functionalizing tantalum to introduce cell‐instructive and stimulus‐responsive properties therefore represents a promising strategy for advancing multifunctional implant systems [32]. Importantly, the intrinsic affinity of tantalum toward oxide formation provides a unique chemical basis for constructing integrated oxide‐based functional interfaces. In our strategy, the laser‐induced Ta_2_O_5_ intermediate layer serves as a reactive platform for Li^+^‐induced conversion into lithium tantalate (LiTaO_3_, LTO), enabling intimate chemical integration between the piezoelectric phase and Ta substrate rather than relying on mechanically attached coatings. Benefiting from its stable crystal structure, excellent chemical stability, and favorable biocompatibility, LiTaO_3_ represents an attractive piezoelectric phase for constructing mechanically responsive biointerfaces. However, whether an implant interface can actively utilize physiological mechanical stimuli to generate bioelectrical cues and regulate the complex interactions among immune, vascular, and osteogenic processes remains largely unexplored. Therefore, developing a force‐electric responsive implant surface represents a promising approach to bridge mechanical stimulation with biological regeneration. Herein, we first report a multifunctional force‐electric responsive Ta implant with piezoelectric LiTaO_3_ nanotentacles fabricated via innovatively combining laser nanofabrication and Li^+^‐induced in situ reaction (Figure 1). Compared with previous piezoelectric coatings, the present design introduces a biomimetic force‐electric nanotentacle interface that not only provides nanoscale structural anchoring but also actively converts external mechanical stimulation into localized bioelectrical cues. This dynamic biointerface simultaneously regulates cellular bioenergetics and multicellular crosstalk, thereby orchestrating immunomodulation‐mediated angiogenesis and osteogenesis for vascularized osseointegration. The novel force‐electric Ta implant endows the real‐time controllable regulation of multifunctional tissue regeneration for using as patient‐personalized load‐bearing osteoarticular prostheses and dental implants.

## 2 Results and Discussion

### 2.1 Construction of Biomimetic Force‐Electric Nanotentacles on Ta

To construct a robust force‐electric response of tantalum implant capable of reliable bioelectrical activation, the laser nanofabrication and Li^+^‐induced in situ reaction processes on Ta substrates were systematically optimized. Initially, laser nanocrystallization induces the formation of tantalum oxide nanostructure coatings on the surface of the tantalum substrate. Laser nanofabrication was performed under different laser powers (25%, 30%, 35%, and 45%) to regulate the surface morphology of the Ta substrate. As shown in Figure S1, increasing laser power led to a gradual evolution from a relatively smooth surface to well‐developed micro‐nanostructures. At low power (25%), only limited melting traces were observed, whereas more uniform nanoparticle‐like emerged at 30% and 35%. In contrast, excessive roughening accompanied by local densification occurred at 45%. Cell compatibility assays (Figure S2) further indicated that the surface fabricated at 30% laser power exhibited the most favorable cytocompatibility, and this condition was therefore selected for subsequent experiments. X‐ray diffraction (XRD) and X‐ray photoelectron spectroscopy (XPS) analyses of the laser‐induced intermediate oxide nanolayer (L/Ta) confirmed the formation of a thin and uniform δ‐Ta_2_O_5_ nanocrystalline layer, with Ta^5^^+^ as the dominant valence state (Figures S3 and S4). Subsequently, these coatings undergo chemical reactions in a lithium solution, resulting in the formation of a dense lithium tantalate nanostructure coating. The growth behavior of LiTaO_3_ on the oxidized Ta surface was subsequently optimized by adjusting the hydrothermal temperature (120, 180, and 240 °C) and reaction time (12–24 h) (Figure S5). This metastable oxide nanolayer, enriched in oxygen vacancies and surface hydroxyl groups, functions not only as a Ta^5^^+^ source but also as an efficient diffusion pathway for Li^+^, thereby serving as a critical structural and chemical bridge between the Ta substrate and the LiTaO_3_ piezoelectric layer.

**FIGURE 2:**
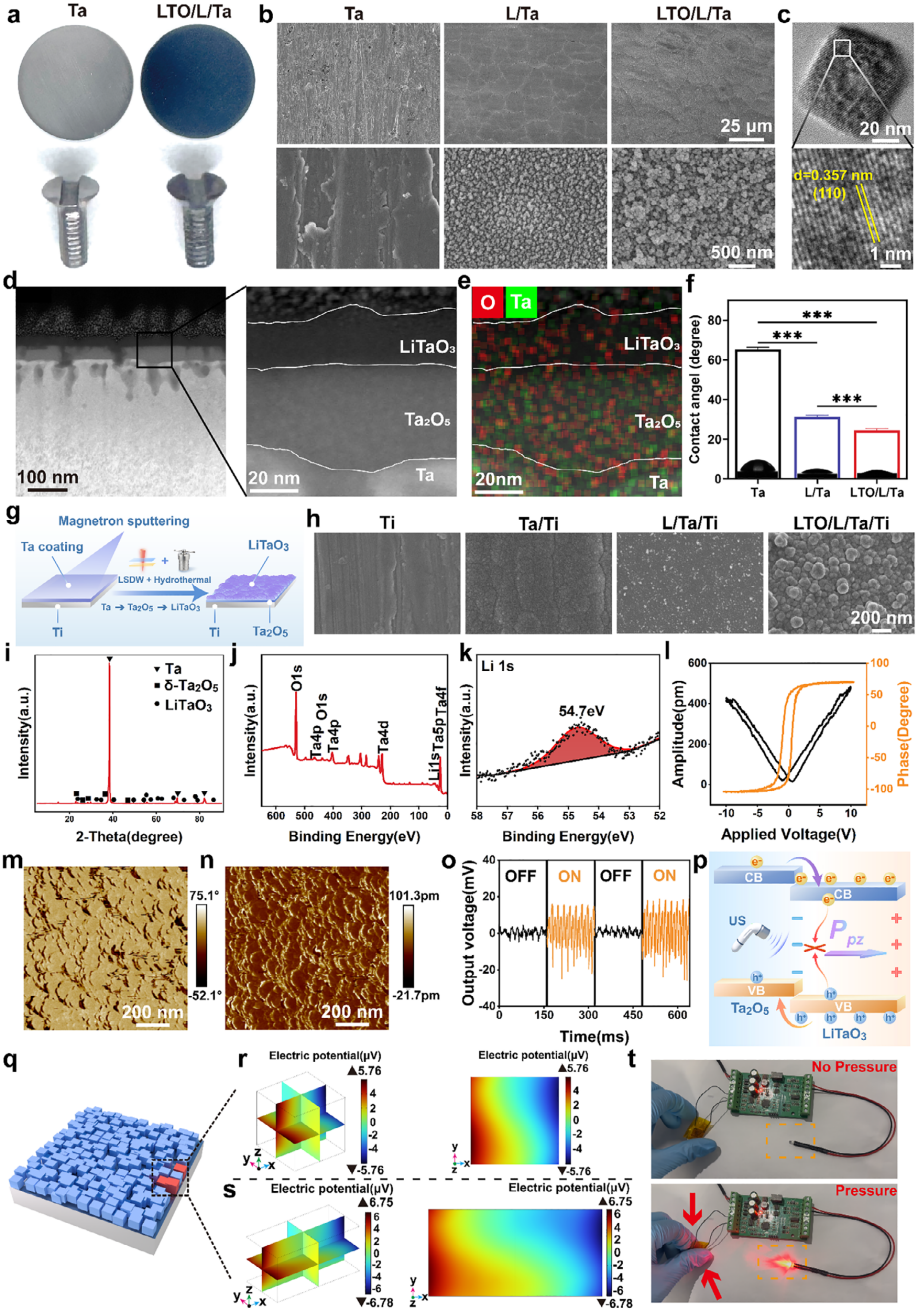
Structural and piezoelectric characterization of force‐electric responsive LTO/L/Ta implant. a) Macroscopic optical images of Ta and LTO/L/Ta. b) Surface SEM morphologies of the three samples. c) HRTEM image of LTO/L/Ta. d) Cross‐sectional TEM image of the interface. e) EDS elemental mapping images. f) Comparison of water contact angles among the three samples. g) Schematic illustration of the fabrication process of the LiTaO_3_ piezoelectric coating constructed on a Ti substrate via magnetron‐sputtered Ta. h) Corresponding SEM image. i) XRD pattern of LTO/L/Ta. j) XPS survey spectrum of LTO/L/Ta. k) High‐resolution XPS spectrum of Li 1s for LTO/L/Ta. l) Piezoelectric displacement–voltage curve of LTO/L/Ta measured by PFM. m) PFM amplitude image of LTO/L/Ta. n) PFM phase image of LTO/L/Ta. o) Piezoelectric output signal of LTO/L/Ta. p) Schematic illustration of the band structure and carrier separation mechanism of LTO/L/Ta under ultrasonic stimulation. q) Three‐dimensional finite element model of force‐electric LiTaO_3_ anchored on a tantalum substrate. r) Simulated electric potential distribution for single LiTaO_3_ nanoparticle under ultrasound stimulation, where the left panel shows a three‐dimensional simulated potential distribution and the right panel shows a *x*–*y* section simulated potential distribution. s) Simulated electric potential distribution for two identical LiTaO_3_ nanoparticles in contact under ultrasound stimulation, where the left panel shows a three‐dimensional simulated potential distribution and the right panel shows a *x*‐*y* section simulated potential distribution. t) Schematic illustration of the electrical response of LTO/L/Ta under mechanical stimulation. The data in (f) is presented as mean ± SEM, *n* = 3. *p*‐values are calculated using one‐way ANOVA followed by Tukey's post hoc test, *p < 0.05, **p < 0.01, ***p < 0.001; ns indicates no significant difference.

Following sequential laser nanofabrication and Li^+^‐induced in situ reaction, the samples exhibited a pronounced color change from metallic silver to deep blue (Figure 2a and Figure S6), reflecting substantial reconstruction of surface chemistry and optical properties. Scanning electron microscopy (SEM) observations (Figure 2b) revealed a smooth surface for pristine Ta, a uniformly distributed nanoparticle morphology after laser oxidation (L/Ta), and a dense LiTaO_3_ composite interface after lithium ion induced in‐situ reaction (LTO/L/Ta). These results indicate that lithium‐ion incorporation markedly alters the oxidation kinetics and facilitates homogeneous interface growth. The structural features of the interface were further elucidated by transmission electron microscopy (TEM) analysis. High‐resolution TEM (HRTEM) images displayed clear lattice fringes with an interplanar spacing of ≈0.357 nm, corresponding to the (110) plane of LiTaO_3_ (Figure 2c). Cross‐sectional TEM combined with elemental mapping revealed a well‐defined trilayer architecture consisting of the Ta substrate, an intermediate δ‐Ta_2_O_5_ layer, and an outer LiTaO_3_ functional layer, with intimate interfacial bonding between adjacent layers (Figure 2d,e and Figure S7). Surface physicochemical properties were further assessed using atomic force microscopy (AFM) and wettability measurements. AFM results showed a pronounced increase in surface roughness for LTO/L/Ta compared with pristine Ta (Figure S8). Consistently, water contact angle measurements (Figure 2f) demonstrated significantly enhanced hydrophilicity for both L/Ta and, more prominently, LTO/L/Ta, which can be attributed to surface hydroxylation and Li^+^‐mediated surface regulation. Such enhanced hydrophilicity is beneficial for interfacial bioactivity, as the wettability of biomaterials has been widely recognized to regulate protein adsorption and subsequent cell adhesion processes [33].

Phase composition and chemical states of the surface were systematically analyzed using XRD, Raman spectroscopy, and XPS. XRD patterns of LTO/L/Ta exhibited multiple characteristic diffraction peaks corresponding to crystalline LiTaO_3_ (Figure 2i), which were further corroborated by Raman spectra (Figure S9) [34]. Simultaneously proving the existence of the intermediate layer δ‐Ta_2_O_5_. XPS survey and high‐resolution spectra (Figure 2j,k and Figure S10) revealed dominant Ta^5^^+^ states and characteristic Li–O bonding, with a distinct Li 1s peak at 54.7 eV, confirming the successful formation of LiTaO_3_ [35]. The universality of this laser‐assisted strategy was further validated by fabricating LiTaO_3_ interface on Ti substrates through magnetron‐sputtered Ta interlayers (Figure 2g,h and Figures S14–S25), as well as directly on Ta substrates via an air‐oxidation–hydrothermal strategy (Figures S11–S13). These results collectively confirm the robustness and general applicability of the fabrication approach. The selection of LiTaO_3_ is also advantageous for the present design because of its excellent physicochemical stability and favorable biocompatibility. More importantly, the Li^+^‐induced in situ reaction enables intimate integration of the piezoelectric phase with the laser‐textured Ta substrate, providing a structurally robust interface for efficient force‐electric conversion under mechanical stimulation. The non‐centrosymmetric crystal structure of LiTaO_3_ provides the structural origin of its intrinsic piezoelectricity, enabling stress‐induced polarization under external mechanical stimulation [36]. Accordingly, the piezoelectric properties of the LTO/L/Ta interface were further evaluated by piezoresponse force microscopy (PFM). Typical piezoelectric behavior was observed, including a butterfly‐shaped amplitude–voltage response and clear phase switching (Figure 2l,m), indicative of strong force‐electric coupling. Under periodic ultrasonic stimulation, the surface generated stable and reversible output signals (Figure 2o), demonstrating efficient mechanical‐to‐electrical energy conversion. This response was further validated by a simple external circuit test: no LED emission was observed in the absence of external force, whereas instantaneous mechanical pressing triggered stable and repeatable LED illumination (Figure 2t), directly confirming effective electrical signal generation.

Based on these observations, a carrier separation and interfacial electrical mechanism under ultrasonic stimulation was proposed (Figure 2p). In this model, piezoelectric polarization induces an internal potential gradient at the Ta_2_O_5_/LiTaO_3_ interface, thereby driving directional charge separation and transport. To elucidate the bioelectrical activation mechanism of the LTO/L/Ta interface under ultrasound stimulation, finite element simulations were performed. Three‐dimensional finite element model of LiTaO_3_ nanoparticles anchored on a tantalum substrate is shown in (Figure 2q). Finite element simulations were conducted to clarify the force‐electric response of the LTO/Ta surface under ultrasonic pressure (140 kPa). As shown in Figure 2r, a single LiTaO_3_ particle generated a clear dipolar potential distribution (−5.76 to +5.76 µV), confirming effective stress‐induced polarization. When two particles were in contact (Figure 2s), the electric field became spatially heterogeneous with increased local potential (−6.78 to +6.75 µV), indicating that interparticle mechanical coupling amplifies polarization intensity and reshapes charge distribution. In addition, the configuration with size‐mismatched nanoparticles is presented in Figure S26, where further field asymmetry is observed. These results demonstrate that nanoscale particle packing enhances local electric field gradients beyond intrinsic piezoelectricity, providing a structural basis for efficient bioelectrical stimulation under stimulation. In parallel, nano‐scratch tests (Figure S27) revealed higher critical loads and lower friction coefficients for LTO/L/Ta, indicating enhanced which can be attributed to the dense and continuous multilayer architecture (Figure S28) [37]. Overall, this laser nanofabrication and Li^+^‐induced in situ reaction processes enables the construction of a LiTaO_3_ force‐electric nanotentacles on Ta substrates, simultaneously improving surface physicochemical properties while imparting robust piezoelectric functionality. This surface therefore provides a solid foundation for subsequent bio‐interfacial regulation and implant related applications.

### 2.2 Osteoimmunomodulatory and Vascularized Osseointegration In Vivo Evaluation

**FIGURE 3:**
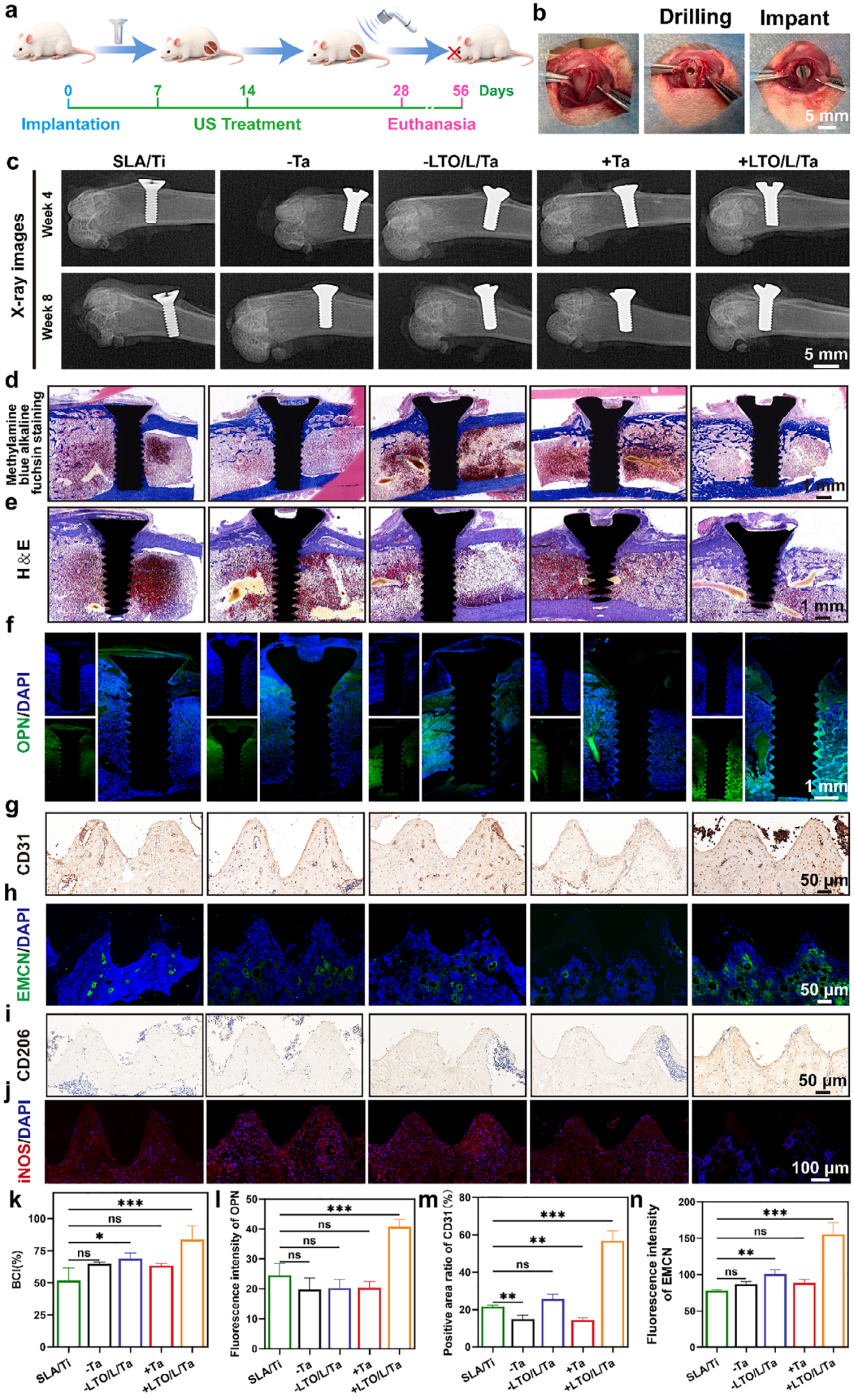
In vivo evaluation of osteoimmunomodulatory and vascularized osseointegration enabled by a force‐electric‐responsive LTO/L/Ta implant. a) Schematic diagram of the animal experimental design. b) Illustration of the surgical procedure. c) X‐ray images of different treatment groups at 4 and 8 weeks post‐operation. d) Hard tissue sections stained with methylene blue/basic fuchsin. e) H&E staining of hard tissue sections. f) Immunofluorescence staining for OPN in bone tissue. g) Immunohistochemical staining for CD31. h) Immunofluorescence staining for EMCN. i) Immunohistochemical staining for CD206. j) Immunofluorescence staining for iNOS. k) Quantitative analysis of bone–implant contact (BIC) in different groups. l) Quantification of OPN fluorescence signal. m) Quantification of CD31‐positive area. n) Quantification of EMCN fluorescence signal. The data in (k), (l), (m), and (n) are presented as mean ± SEM, *n* = 5 for (k); *n* = 3 for (l)–(n). *p*‐values are calculated using one‐way ANOVA followed by Tukey's post hoc test, *p < 0.05, **p < 0.01, ***p < 0.001; ns indicates no significant difference.

The in vivo performance of the piezoelectric LTO/L/Ta surface was systematically evaluated using a rat femoral defect model, with continuous imaging and histological analyses conducted at 4 and 8 weeks after implantation and ultrasound treatment (Figure S29). This approach enabled direct observation of how the ultrasound‐activated piezoelectric LTO/L/Ta surface establishes a favorable local microenvironment, promotes coordinated angiogenesis and osteogenesis, and ultimately enhances bone–implant integration. Comparative analyses with uncoated Ta and SLA/Ti surfaces allowed evaluation of both early and late‐stage osteogenic and angiogenic responses. Imaging and histological analyses performed at 4 and 8weeks postsurgery (Figure 3a). The surgical procedure was stable and reproducible (Figure 3b), providing a reliable foundation for subsequent assessment of tissue repair. To objectively benchmark the performance of the piezoelectric LTO/L/Ta functionalized surface, the widely used sandblasted and acid‐etched titanium surface (SLA/Ti) was employed as a control group, with its typical morphology shown in Figure S30, enabling direct comparison of in vivo osteogenic outcomes. X‐ray imaging (Figure 3c) revealed that, at 4 weeks, the +LTO/L/Ta group already exhibited clear new bone formation surrounding the implant, whereas control and unmodified groups retained extensive radiolucent areas. By 8 weeks, the +LTO/L/Ta group had developed continuous, dense bone within the defect region, with indistinct implant‐bone boundaries, indicating markedly enhanced osseointegration. Although X‐ray imaging provides two‐dimensional visualization of implant placement, it was combined with subsequent histological and histomorphometric analyses to evaluate peri‐implant bone formation and bone–implant integration.

Histological analyses were highly consistent with these imaging results. Toluidine Blue/Basic Fuchsin staining (Figure 3d) demonstrated abundant mature lamellar bone around the implant in the +LTO/L/Ta group, characterized by dense trabecular arrangement and tight bone–implant integration; in contrast, mature bone formation was comparatively limited in control groups. Concurrently, bone–implant contact (BIC) ratios (Figure 3k) were significantly higher than those in other groups. Hematoxylin and eosin (H&E) staining (Figure 3e) further confirmed this trend, showing more continuous and mature bone tissue structures in the +LTO/L/Ta group. OPN immunofluorescence staining (Figure 3f) revealed the strongest signal in the +LTO/L/Ta group, with quantitative analysis (Figure 3l) confirming the highest OPN expression, reflecting significantly enhanced osteogenic activity.

Beyond osteogenesis, the piezoelectric LTO/L/Ta functionalized surface also elicited a robust angiogenic response at the bone–implant interface. CD31 immunohistochemical staining (Figure 3g) and EMCN immunofluorescence staining (Figure 3h) revealed a richer and more mature vascular network in the +LTO/L/Ta group compared with all controls. Quantitative analyses confirmed that both CD31 positive area (Figure 3m) and EMCN fluorescence intensity (Figure 3n) reached their highest levels in this group, indicative of enhanced endothelial activity and type H vessel formation. Notably, these osteogenic and angiogenic effects exhibited clear temporal progression. As early as 4 weeks post‐implantation, significantly higher new bone volume and vascular density were already observed in the +LTO/L/Ta group (Figures S31–S39), while further maturation and vascular complexity developed by 8 weeks. Collectively, these findings demonstrate that the ultrasound‐activated piezoelectric surface establishes a favorable local microenvironment that initiates early bone repair and sustains the coupled progression of osteogenesis and angiogenesis, ultimately leading to markedly improved osseointegration. To further validate the immunomodulatory effect of the LTO/L/Ta interface within the bone–implant microenvironment, macrophage polarization was evaluated in peri‐implant tissues collected from rat femoral defects at 4 weeks after implantation. CD206 immunohistochemical staining revealed relatively weak expression in the SLA/Ti, ‐Ta, ‐LTO/L/Ta, and +Ta groups, whereas the +LTO/L/Ta group exhibited markedly enhanced CD206‐positive staining around the implant interface (Figure 3i), indicating enhanced recruitment or polarization of reparative M2‐like macrophages. Conversely, iNOS immunofluorescence staining showed abundant positive signals in the SLA/Ti and ‐Ta groups, while reduced iNOS expression was observed in the ‐LTO/L/Ta and +Ta groups. Notably, the +LTO/L/Ta group exhibited the lowest iNOS signal among all groups (Figure 3j), demonstrating effective suppression of pro‐inflammatory macrophage activation. Quantitative analysis further confirmed these observations, showing a significantly increased CD206‐positive area ratio and reduced iNOS fluorescence intensity in the +LTO/L/Ta group compared with all control groups (Figure S40). Collectively, these results confirm that ultrasound‐activated LTO/L/Ta interfaces establish a more favorable immune microenvironment by promoting M2‐like polarization and attenuating inflammatory responses at the bone–implant interface. Importantly, histological examination of major organs, including the heart, liver, spleen, lung, and kidney (Figure S41), revealed no evident pathological abnormalities across all groups, indicating satisfactory systemic biosafety of the LTO/L/Ta implant. To further assess the structural stability of the functionalized implant after implantation under physiological conditions, LTO/L/Ta screws were retrieved after 8 weeks of implantation in the rat femoral model. Optical images revealed no obvious peeling, cracking, or macroscopic structural damage after implantation (Figure S42). Moreover, SEM characterization demonstrated that the nanoscale surface morphology of the functional interface was largely preserved after implantation (Figure S43). Importantly, the explanted implants retained reversible piezoelectric voltage responses under mechanical stimulation, although a slight reduction in output amplitude was observed compared with pristine samples, confirming the sustained force‐electric conversion capability of the LTO/L/Ta interface after long‐term implantation (Figure S44, Supporting). In conclusion, the in vivo results clearly demonstrate that the piezoelectric LTO/L/Ta functionalized surface synergistically enhances bone formation and vascularization under LIPUS, thereby substantially improving implant osseointegration. This “osteogenesis–angiogenesis” coupling effect, mediated through local microenvironment regulation, provides compelling experimental evidence for the rational design of high‐performance bone repair materials.

### 2.3 In Vivo Immunomodulation and the Molecular Mechanism

**FIGURE 4:**
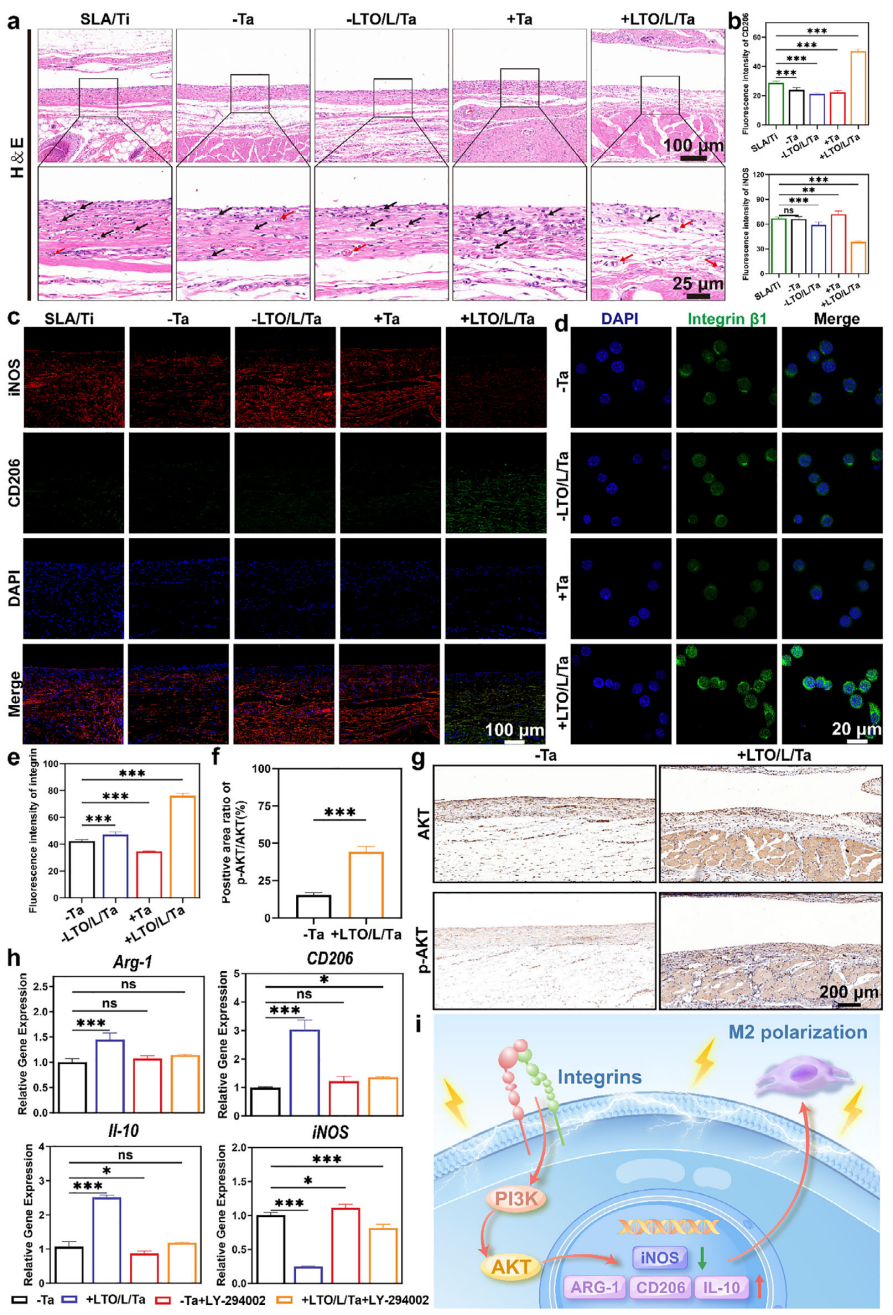
In vivo immunomodulatory validation of force‐electric responsive LTO/L/Ta implant (dorsal subcutaneous implantation model). a) Representative H&E staining images (top: low magnification; bottom: high magnification). Black arrows indicate areas of inflammatory cell accumulation; red arrows indicate the area of angiogenesis. b) Quantitative analysis of fluorescence intensity for M2 marker CD206 and M1 marker iNOS in tissue sections. c) Representative immunofluorescence images showing the expression distribution of iNOS (red), CD206 (green), and nuclei (DAPI, blue) across different groups. d) Immunofluorescence staining of macrophage integrins (green) to visualize adhesion signal activation on different material surfaces. e) Quantification of integrin fluorescence intensity. f) Quantitative analysis of the proportion of p‐AKT/AKT positive area. g) Immunohistochemical staining of AKT and p‐AKT implanted in skin tissues. h) qRT‐PCR analysis of immune‐related genes (*iNOS*, *Il‐10*, *Arg‐1*, *CD206*) in tissues from different treatment groups. i) Schematic illustration of the mechanism by which macrophage polarization is regulated. The data in (b), (e), (f), and (h) are presented as mean ± SEM, *n* = 5 for (b); *n* = 3 for (e), (f), and (h). *p*‐values are calculated using one‐way ANOVA followed by Tukey's post hoc test, *p < 0.05, **p < 0.01, ***p < 0.001; ns indicates no significant difference.

The immunomodulatory capacity of the piezoelectric LTO/L/Ta functionalized surface under LIPUS was evaluated in vivo using a dorsal subcutaneous implantation model, enabling the assessment of early inflammatory responses and macrophage phenotypes (Figure S45). The dorsal subcutaneous implantation model was employed as a complementary approach to evaluate the intrinsic immunomodulatory properties of the implant surface. Although this model does not completely reproduce the bone–implant microenvironment, it minimizes the influence of bone injury and remodeling processes, enabling a more direct evaluation of material‐induced inflammatory responses and macrophage polarization. H&E staining (Figure 4a) revealed pronounced inflammatory cell infiltration, loose tissue architecture, and some extravasation of red blood cells around SLA/Ti and Ta implants. In contrast, tissue surrounding the LTO/L/Ta group was more compact with substantially fewer inflammatory cells. Upon additional LIPUS stimulation (+LTO/L/Ta), inflammation was further attenuated, tissue layers remained more intact, and cellular organization appeared more orderly, indicating that the piezoelectric LTO/L/Ta functionalized surface effectively mitigates early inflammatory responses and promotes the establishment of a reparative microenvironment in vivo. Considering the central role of macrophages in immune regulation, M1/M2 phenotypic shifts were further assessed by immunofluorescence. Dual staining for iNOS and CD206 (Figure 4c) showed strong iNOS and weak CD206 signals in SLA/Ti and Ta groups, indicative of a pro‐inflammatory local environment. By comparison, the +LTO/L/Ta group exhibited the lowest iNOS and the highest CD206 signals, suggesting increased M2 polarization. Quantitative analysis (Figure 4b) confirmed a significant increase in CD206 and a corresponding decrease in iNOS in the +LTO/L/Ta group, demonstrating that the piezoelectric‐activated LTO/L/Ta surface establishes a reparative, anti‐inflammatory immune microenvironment.

Mechanistic insights were obtained by examining integrin signaling in macrophages. Immunofluorescence analysis (Figure 4d) revealed markedly enhanced integrin expression on the piezoelectric LTO/L/Ta functionalized surface, which was further amplified under ultrasound stimulation (+LTO/L/Ta), as supported by quantitative measurements (Figure 4e). Further in vivo immunohistochemistry demonstrated significant upregulation of AKT and its activated phosphorylated form p‐AKT in the +LTO/L/Ta group (Figure 4g), with quantitative analysis of p‐AKT/AKT‐positive area (Figure 4f) confirming substantial activation. This finding supports a central role for integrin‐mediated PI3K–AKT signaling in macrophage modulation by the piezoelectric LTO/L/Ta functionalized surface. Consistently, gene expression analysis (Figure 4h) revealed that the +LTO/L/Ta group significantly downregulated pro‐inflammatory *iNOS* while upregulating M2‐associated genes including *Arg‐1*, *CD206*, and *Il‐10*. Application of the PI3K inhibitor LY294002 partially or completely reversed these effects, supporting the involvement of PI3K–AKT signaling in piezoelectric LTO/L/Ta functionalized surface‐mediated immune reprogramming. Integrin β1 serves as a critical mechanosensitive receptor in macrophages, linking extracellular mechanical cues to intracellular signaling responses through adhesion‐associated pathways. Previous studies have demonstrated that integrin engagement can activate focal adhesion‐associated molecules, including FAK/Src, which subsequently promote PI3K recruitment and AKT phosphorylation, thereby regulating macrophage polarization and inflammatory responses [20, 38, 39]. Consistently, our results showed that LTO/L/Ta‐mediated force‐electric stimulation enhanced Integrin β1 expression, accompanied by increased PI3K/AKT activation and M2‐like polarization. These findings suggest that Integrin β1‐associated mechanotransduction may participate in force‐electric regulation of macrophage phenotype through the PI3K/AKT signaling axis (Figure 4i), thereby contributing to the establishment of an immune microenvironment favorable for subsequent osteogenesis and angiogenesis.

### 2.4 Osteogenic Differentiation Ability and Its Molecular Mechanism

Given that bioelectric cues generated by piezoelectric materials under mechanical stimulation have been recognized as critical regulators of stem cell fate [40], we further evaluated whether LIPUS‐triggered piezoelectric activation of the LTO/L/Ta surface could enhance the osteogenic responses of rat bone marrow mesenchymal stem cells (rBMSCs). Prior to the systematic evaluation of the osteogenic effects of the LiTaO_3_ piezoelectric surface, a series of preliminary experiments were conducted to determine appropriate ultrasonic stimulation parameters. CCK‐8 assays combined with osteogenic gene expression analyses in rBMSCs under different ultrasonic conditions showed that 3 W/cm^2^ ultrasonic intensity effectively preserved cell viability while simultaneously promoting osteogenic gene activation (Figures S46–S49). Based on these observations, an ultrasonic stimulation protocol of 3 W/cm^2^ for 5 min was selected for all subsequent experiments.

**FIGURE 5:**
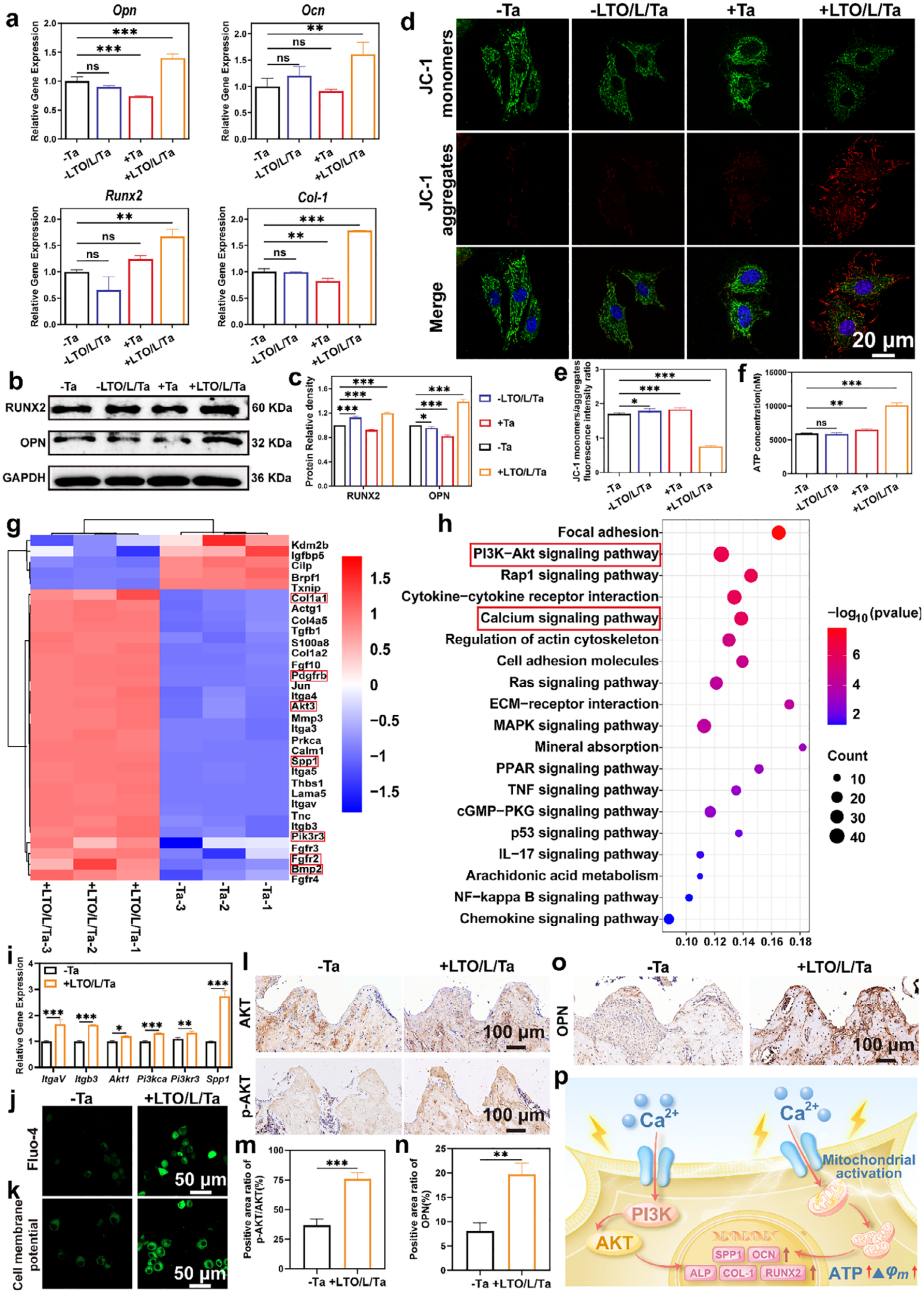
In vitro evaluation of force‐electric responsive LTO/L/Ta implant promoting osteogenic differentiation under LIPUS, as well as multi‐omics and cellular mechanism analysis of regulating osteogenic signaling pathways. a) qRT‐PCR analysis of osteogenesis‐related gene expression (*Opn*, *Ocn*, *Runx2*, and *Col‐1*) after 7 days of culture. b) Western blot analysis of osteogenic marker proteins RUNX2 and OPN. c) Densitometric quantification of RUNX2 and OPN protein expression. d) JC‐1 staining of cells to assess mitochondrial membrane potential (ΔΨm): green fluorescence indicates JC‐1 monomers, and red fluorescence indicates JC‐1 aggregates. e) Quantitative analysis of the fluorescence intensity ratio of JC‐1 monomers to aggregates. f) Intracellular ATP content of cells in different groups. g) Hierarchical clustering heatmap of key differentially expressed genes. h) KEGG pathway enrichment analysis showing significantly activated signaling pathways. i) qRT‐PCR analysis of osteogenesis‐ and signaling‐related genes (*ItgaV*, *Itgb3*, *Akt1*, *Pi3kca, Pi3kr3*, and *Spp1*). j) Intracellular Ca^2^^+^ levels detected by the Fluo‐4 fluorescent probe. k) Cell membrane potential assessed using the DiBAC4(3) fluorescent probe. l) Immunohistochemical staining of AKT and p‐AKT in implanted bone tissue. m) Quantification of positive staining areas for p‐AKT/AKT. n) Quantification of positive staining areas for OPN. o) Immunohistochemical staining of OPN in implanted bone tissue. p) Schematic illustration of the proposed mechanism by which LTO/L/Ta promotes osteogenic differentiation under ultrasonic stimulation. The data in (a), (c), (e), (f), (i), (m), and (n) are presented as mean ± SEM, *n* = 3 for (a), (c), (e), (f), and (i); *n* = 5 for (m) and (n). *p*‐values are calculated using one‐way ANOVA followed by Tukey's post hoc test, *p < 0.05, **p < 0.01, ***p < 0.001; ns indicates no significant difference.

The cytocompatibility of the LTO/L/Ta surface was further confirmed by Live/Dead staining and CCK‐8 assays, which revealed higher cell coverage and sustained proliferation under ultrasonic stimulation (Figures S50 and S51). In parallel, cytoskeletal staining showed that cells cultured on LTO/L/Ta displayed enhanced spreading with well‐developed actin stress fibers, together with increased spreading area and more mature focal adhesion formation (Figures S52 and S53). The protruding nanoscale LiTaO_3_ domains provide abundant anchoring sites that facilitate cell adhesion and focal adhesion maturation. Meanwhile, these mechanoresponsive nanostructures can convert mechanical stimulation into localized electrical cues under ultrasonic activation, resembling tentacle‐like electromechanical units at the biointerface. We therefore refer to these structures as “force‐electric nanotentacles.” Osteogenic differentiation was subsequently evaluated at both the gene and protein levels. After 7 days of induction, qRT‐PCR analysis revealed significant upregulation of osteogenic markers, including *Runx2*, *Alp*, *Col‐1*, and *Opn*, in cells cultured on LTO/L/Ta under ultrasonic stimulation (Figure 5a). These pro‐osteogenic effects were maintained at later stages, as evidenced by enhanced ALP staining, increased mineral deposition observed by Alizarin Red S staining, and enhanced osteogenic gene expression at day 14 (Figures S54 and S55). Consistently, immunofluorescence staining and Western blot analysis demonstrated elevated RUNX2 and OPN protein expression in the +LTO/L/Ta group (Figure 5b,c; Figures S58 and S59), confirming effective translation of transcriptional activation into functional protein expression. In parallel, PCR results demonstrated that the laser‐induced oxide nanolayer possessed mild osteogenic potential (Figures S60 and S61). However, this effect was substantially amplified after lithium ion induced in situ reaction, suggesting a critical role of piezoelectric activation in functional enhancement.

Given the pivotal role of mitochondrial bioenergetics in supporting osteogenic commitment and differentiation [41], we next investigated whether the force‐electric stimulation generated by the LTO/L/Ta interface could modulate mitochondrial function in BMSCs. To examine the influence of the LiTaO_3_ surface on cellular bioenergetic status, mitochondrial membrane potential (ΔΨm) was assessed using JC‐1 staining (Figure 5d). Compared with the control group (‐Ta), cells cultured on LTO/L/Ta under ultrasound low intensity ultrasound (LIPUS) exhibited markedly enhanced red fluorescence accompanied by reduced green fluorescence, indicating an elevated ΔΨm. Quantitative analysis further confirmed a significant decrease in the JC‐1 monomer‐to‐aggregate ratio in the LTO/L/Ta group (Figure 5e). Consistent with these findings, intracellular ATP levels were significantly increased on +LTO/L/Ta compared with both Ta group (Figure 5f), demonstrating that piezoelectric stimulation effectively enhanced mitochondrial bioenergetic activity and cellular energy supply. Beyond serving as a metabolic indicator, ATP production represents an important bioenergetic response connecting force‐electric stimulation with osteogenic activity. Osteogenic differentiation is an energy‐intensive process requiring sufficient metabolic support for extracellular matrix (ECM) synthesis, collagen deposition, and subsequent mineralization. Therefore, the enhanced ATP generation induced by the LTO/L/Ta interface under LIPUS stimulation may provide the necessary energetic foundation for osteogenic differentiation and maturation of rBMSCs. To further elucidate the molecular mechanisms underlying LTO/L/Ta‐induced osteogenesis under LIPUS, transcriptomic analysis was performed. A total of 1378 differentially expressed genes were identified between the +LTO/L/Ta and Ta groups (Figure S62). Hierarchical clustering analysis revealed pronounced upregulation of gene clusters associated with osteogenesis, cell adhesion, cytoskeletal organization, and intracellular signal transduction (Figure 5g). KEGG pathway enrichment analysis further demonstrated significant activation of pathways closely related to mechanotransduction and osteogenic differentiation, including the PI3K–AKT signaling pathway, calcium signaling pathway, ECM–receptor interaction, focal adhesion, and Rap1 signaling pathways (Figure 5h). These pathway‐level alterations were further supported by GO enrichment analysis, which showed enhanced biological processes related to calcium signaling, ECM organization, cell migration, and mechanosensitive responses (Figure S63). Several representative genes involved in mechanotransduction and osteogenesis, including *Akt1*, *Pi3kca*, and *Spp1* (OPN), were subsequently validated by qRT‐PCR (Figure 5i), in good agreement with the transcriptomic results. Given the prominent enrichment of calcium‐related signaling pathways, intracellular Ca^2^^+^ dynamics were further examined. Fluo‐4 staining revealed significantly elevated intracellular Ca^2^^+^ levels in cells cultured on LTO/L/Ta under ultrasonic stimulation (Figure 5j; Figures S64a and S65). In parallel, membrane potential measurements using DiBAC4(3) demonstrated enhanced cellular electrical activity in the +LTO/L/Ta group (Figure 5k; Figures S64b and S66), suggesting that piezoelectric stimulation directly modulates cellular electrophysiological behavior. To distinguish the effect of ultrasound stimulation from piezoelectric activation, additional control groups were evaluated. As shown in Figure 5j,k and Figure S64, the +Ta group exhibited only limited changes in intracellular Ca^2^^+^ levels and membrane potential compared with ‐Ta, whereas pronounced Ca^2^^+^ influx and membrane depolarization were observed in the +LTO/L/Ta group compared with ‐LTO/L/Ta under LIPUS stimulation. These results indicate that the enhanced bioelectrical response originates from the activation of the LiTaO_3_ piezoelectric interface rather than ultrasound stimulation alone. Immunohistochemical analysis showed that compared with ‐Ta group, the expression of p‐AKT in the +LTO/L/Ta group was significantly upregulated (Figure 5l), and the corresponding quantitative analysis of p‐AKT/AKT in the positive area further confirmed this conclusion (Figure 5m). The positive staining area and quantitative analysis of SPP1 (OPN) in the +LTO/L/Ta group were significantly increased (Figure 5n,o). Collectively, these findings suggest that the piezoelectric LTO/L/Ta functionalized surface integrates LiTaO_3_ nanotentacles with piezoelectric microelectrical stimulation and bioenergetic regulation, thereby synergistically enhancing Ca^2^^+^ influx, integrin‐mediated adhesion signaling, and PI3K–Akt pathway activation. Based on these findings, a mechanistic model is proposed (Figure 5p), in which mechanical stimulation is converted into piezoelectric electrical signals that activate calcium channels and mitochondrial function, which may subsequently contribute to increased ATP production and subsequent activation of osteogenic transcriptional programs. This multilevel “electrical–calcium–energy–transcription” coupling mechanism ultimately drives robust osteogenic differentiation.

### 2.5 Evaluation of Immunomodulatory Effects and Immune‐Mediated Osteogenesis and Angiogenesis via Paracrine Signaling

Macrophages play a pivotal role in orchestrating the early response and determining the trajectory of subsequent tissue regeneration. The timely transition from a pro‐inflammatory M1 phenotype to a reparative M2 phenotype is essential for resolving inflammation and enabling angiogenesis and osteogenesis [42]. To investigate whether piezoelectric activation of the piezoelectric LTO/L/Ta functionalized surface can actively reprogram macrophage behavior, we systematically evaluated phenotype markers, cytokine secretion, and cytoskeletal organization under LIPUS stimulation, with particular attention to the role of force‐electric induction in creating surface charges that direct macrophage polarization.

**FIGURE 6:**
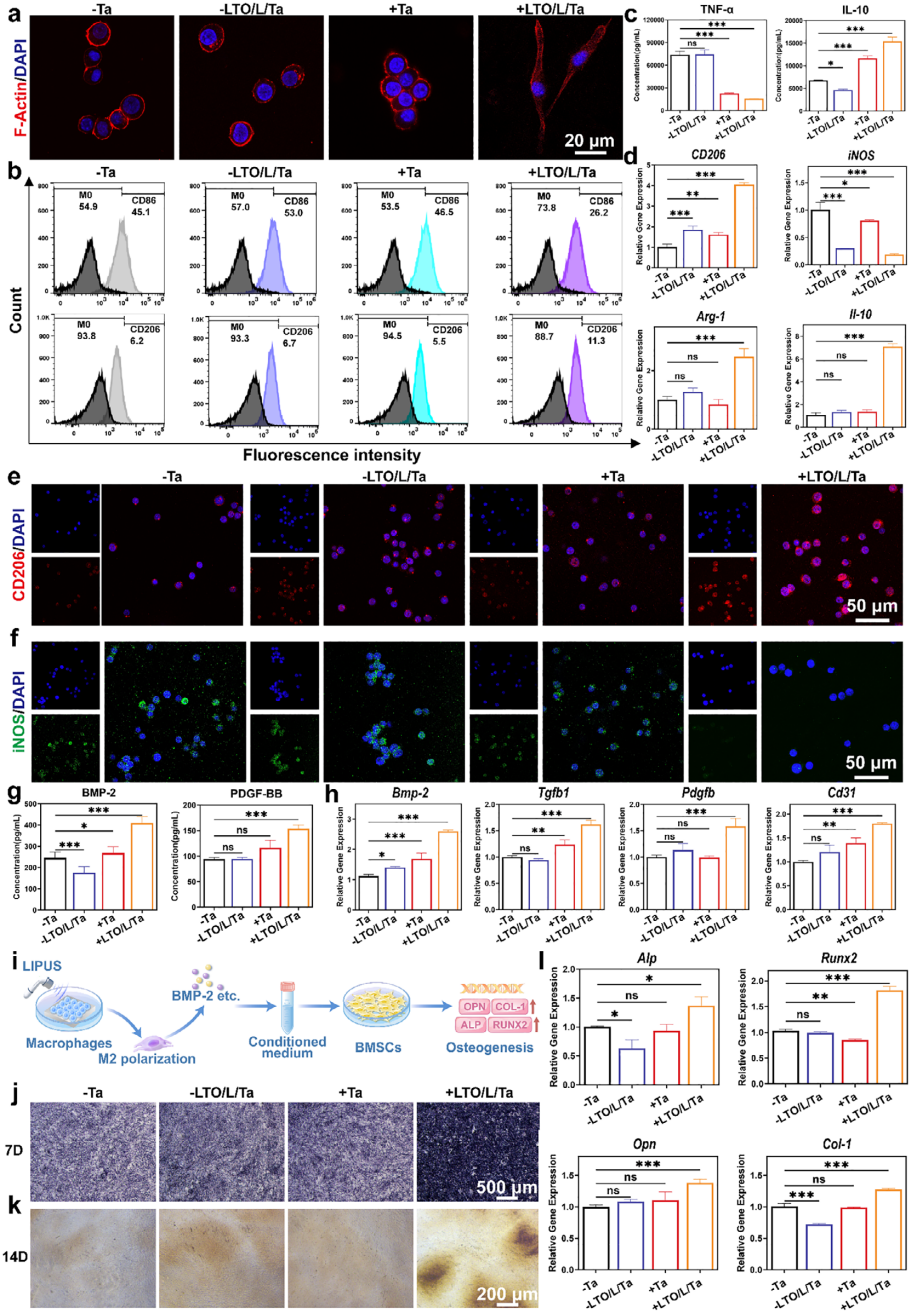
Force‐electric responsive LTO/L/Ta interface orchestrates M2 macrophage polarization and osteogenesis–angiogenesis crosstalk under LIPUS stimulation. a) F‐actin/DAPI immunofluorescence staining of RAW 264.7 in different groups. b) Flow cytometry analysis of CD86 (M1 marker) and CD206 (M2 marker) expression. c) ELISA quantification of TNF‐α and IL‐10 secretion. d) qRT‐PCR analysis of M1/M2‐related genes (*iNOS*, *CD206*, *IL‐10*, *Arg‐1*). e,f) Immunofluorescence staining of CD206 and iNOS. g) Concentrations of BMP‐2 and PDGF‐BB secreted by macrophages after treatment on different material surfaces. h) Relative expression levels of osteogenesis‐angiogenesis‐related genes (*Bmp‐2*, *Tgfb1*, *Pdgfb*, *Cd31*) in RAW 264.7. i) Schematic illustration of the paracrine effect experiment with macrophages. j) ALP staining of rBMSCs cultured in conditioned media from different treatments. k) Mineralized nodule staining of rBMSCs. l) Expression analysis of osteogenic genes (*Alp*, *Runx2*, *Opn*, *Col‐1*). The data in (c), (d), (g), (h), and (l) are presented as mean ± SEM, *n* = 3. *p*‐values are calculated using one‐way ANOVA followed by Tukey's post hoc test, *p < 0.05, **p < 0.01, ***p < 0.001; ns indicates no significant difference.

The effects of varying ultrasonic stimulation durations on macrophage phenotypes (Figure S67) were systematically evaluated. M2‐associated markers (*CD206*, *Arg‐1*, and *Il‐10*) exhibited a gradual increase with stimulation durations of 2, 5, and 10 min/day, whereas M1‐associated markers (INOS, *Tnf‐α*) showed a decreasing trend. A daily stimulation condition of 3 W/cm^2^ for 10 min was identified as optimal. Subsequent mechanistic experiments were performed under this optimal stimulation to systematically evaluate material induced immunomodulatory effects. The effects of the piezoelectric LTO/L/Ta functionalized surface on macrophage activity were evaluated using CCK‐8 assays (Figure S68) and Live/Dead staining (Figure S69), both of which demonstrated high cell viability across all groups regardless of piezoelectric activation. Notably, the +LTO/L/Ta group exhibited a slight yet significant increase in proliferation over 1–3 days, indicating that the piezoelectric microenvironment did not induce cytotoxicity and effectively preserved normal cellular metabolic activity. In line with these findings, SEM observations of cell adhesion (Figure S70) revealed that macrophages adopted a spindle‐shaped morphology characteristic of M2 activation in the +LTO/L/Ta group. This morphological shift was further corroborated by F‐actin staining (Figure 6a), suggesting that the piezoelectric potential generated at the material surface actively participates in macrophage immunomodulation. Consistent with the observed morphological changes, flow cytometry analysis showed that, compared with the Ta and the LTO/L/Ta groups, +LTO/L/Ta group significantly reduced the expression of the M1 marker CD86 while markedly increasing the proportion of CD206‐positive cells (Figure 6b). Correspondingly, enzyme‐linked immunosorbent assay (ELISA) measurements revealed a pronounced decrease in the pro‐inflammatory cytokine TNF‐α alongside a substantial increase in the anti‐inflammatory cytokine IL‐10 in the +LTO/L/Ta group (Figure 6c), further supporting a phenotypic shift of macrophages from a pro‐inflammatory toward a regenerative state.

Immunofluorescence staining further confirmed this directed phenotypic transition. Specifically, CD86 fluorescence intensity was significantly attenuated in the +LTO/L/Ta group, whereas CD206 expression was markedly enhanced and iNOS was suppressed (Figure 6e,f), collectively indicating a clear immunoregulatory bias. Quantitative analysis of fluorescence signals (Figure S71) further substantiated these trends. At the genetic level, the +LTO/L/Ta group showed significant downregulation of *iNOS* and *TNF‐α*, accompanied by maximal upregulation of *IL‐10* and *Arg‐1* (Figure 6d), which are hallmark features of an M2‐dominant regenerative immune microenvironment. To further clarify the specific contribution of each material component, supplementary experiments demonstrated that the laser‐oxidized surface alone (Ta_2_O_5_) did not induce significant changes in the expression of macrophage polarization‐related genes (Figure S72). These results indicate that the observed immunomodulatory effects do not arise from the Ta_2_O_5_ layer itself, but are instead specifically mediated by the ultrasound responsive LiTaO_3_ layer, where surface charge generation serves as the critical signal driving macrophage polarization toward a pro‐regenerative phenotype. Overall, the piezoelectric LTO/L/Ta functionalized surface integrates transient piezoelectric potentials with nanoscale surface architectures to robustly guide macrophages toward M2 polarization. Through coordinated regulation of cell adhesion, cytokine secretion, and cytoskeletal organization, this immunomodulatory surface establishes a regenerative immune microenvironment that suppresses inflammation while promoting tissue repair, thereby providing a critical immunological basis for the subsequently observed enhancement of osteogenesis and angiogenesis. The coordinated interplay between the immune microenvironment, bone formation, and angiogenesis is critical for effective tissue regeneration [43]. At the level of the inflammatory microenvironment, key osteogenic–angiogenic regulatory factors exhibited pronounced differences among groups. ELISA analysis revealed that, compared with the uncoated control, both Ta and LTO/L/Ta surfaces significantly enhanced macrophage secretion of BMP‐2 and PDGF‐BB, with the highest levels observed in the +LTO/L/Ta group (Figure 6g), indicating the formation of a paracrine milieu favorable for tissue repair. Consistently, qPCR analysis demonstrated that macrophages exposed to +LTO/L/Ta markedly upregulated the expression of regeneration‐associated genes, including *Bmp‐2*, *Tgfb1*, *Pdgfb*, and *Cd31* (Figure 6h), suggesting activation of a cytokine‐mediated osteo‐vascular signaling axis [44, 45]. The functional consequences of this immunomodulatory paracrine signaling on osteogenesis were further evaluated by applying macrophage‐conditioned media to osteogenic cells. Both ALP staining and Alizarin Red S staining demonstrated that conditioned medium from the +LTO/L/Ta group elicited the most pronounced mineralization response (Figure 6j, k). In parallel, transcriptional analysis showed significant upregulation of osteogenic markers, including *Alp*, *Runx2*, *Opn*, *Col‐1*, under +LTO/L/Ta conditions (Figure 6l). Together, these findings indicate that the reshaped immune microenvironment effectively promotes osteogenic differentiation through enhanced intercellular paracrine signaling. Beyond bone formation, the immunomodulatory surface exerted a similarly strong influence on angiogenic behavior. qPCR analysis demonstrated that conditioned medium derived from the +LTO/L/Ta group significantly increased the expression of angiogenesis‐related genes, including *Emcn*, *Cd31*, and *Vegfa*, in endothelial cells (Figure S73). Functionally, scratch assays revealed accelerated wound closure within 24 h in the +LTO/L/Ta group, as confirmed by quantitative analysis (Figure S74). Consistently, Transwell migration assays showed the highest number of migrated endothelial cells under +LTO/L/Ta conditions (Figure S77), while tube formation staining and quantitative analysis further demonstrated robust enhancement of endothelial activation and capillary‐like network formation (Figures S75 and S76). In summary, the force‐electric response piezoelectric LTO/L/Ta functionalized surface, when activated by LIPUS stimulation, effectively reprograms macrophage immune phenotypes and amplifies osteo‐vascular paracrine signaling. This immune‐mediated crosstalk simultaneously promotes osteogenic differentiation and endothelial tube formation, thereby establishing a highly synergistic regenerative microenvironment. Concurrently, these results elucidate the multilevel interplay among immune regulation, bone formation, and angiogenesis, and underscore the value of the multifunctional force‐electrical responsive tantalum implant for coordinated and complex tissue regeneration.

### 2.6 The Molecular Mechanism of Immune‐Mediated Osteogenic Regulation

**FIGURE 7:**
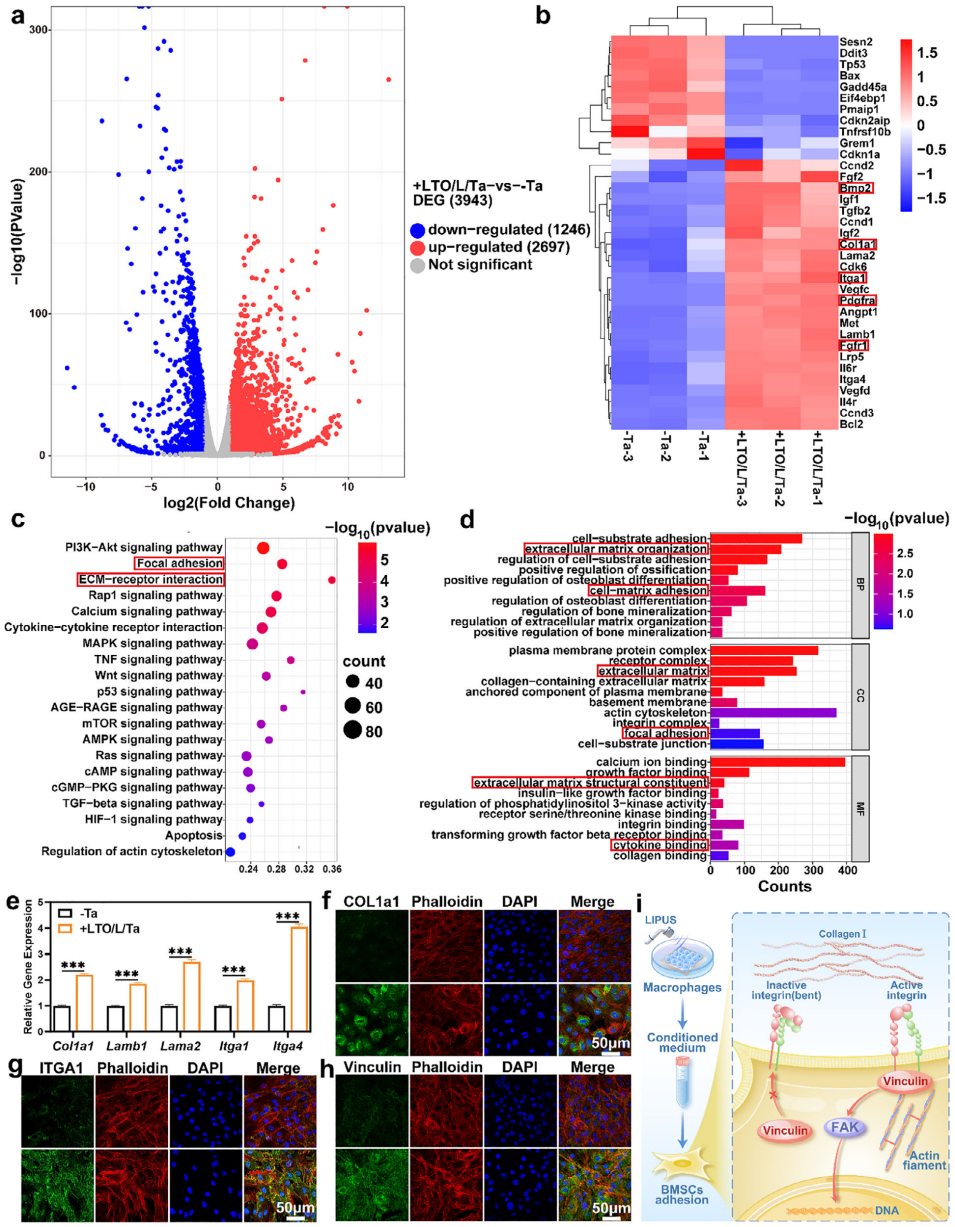
Mechanistic analysis of immune‐osteogenesis crosstalk regulated by force‐electric responsive LTO/L/Ta implant. a) Volcano plot of differentially expressed genes between +LTO/L/Ta and Ta groups: red indicates upregulated genes, blue indicates downregulated genes. b) Hierarchical clustering heatmap of the differentially expressed genes, highlighting ECM‐integrin‐related genes including *Itga1*, *Lama2*, *Col1a1*, *Itga4*, and *Lamb1*. c) KEGG enrichment analysis showing significant enrichment of ECM–receptor interaction, Focal adhesion, PI3K–Akt signaling pathway, Rap1 signaling pathway, and Calcium signaling pathway. d) GO enrichment analysis indicating that the differentially expressed genes are primarily involved in cell–matrix adhesion, extracellular matrix organization, osteoblast differentiation, bone mineralization, and angiogenesis. e) qPCR validation of five ECM‐integrin‐related genes (*Itga1*, *Lama2*, *Col1a1*, *Itga4*, *Lamb1*) after immunomodulation. f) Immunofluorescence staining of COL1A1 (green) and F‐actin (Phalloidin, red). g) Immunofluorescence staining of ITGA1 (green) and F‐actin (Phalloidin, red). h) Immunofluorescence staining of Vinculin (green) and F‐actin (Phalloidin, red). i) Schematic diagram illustrating the signaling mechanisms underlying immune‐osteogenesis crosstalk regulation. RNA sequencing was performed using three biological replicates per group (*n* = 3). Differentially expressed genes were identified based on adjusted *p* < 0.05 and |log_2_FC| ≥ 1. The data in (e) is presented as mean ± SEM, *n* = 3. *p*‐values are calculated using one‐way ANOVA followed by Tukey's post‐hoc test, *p < 0.05, **p < 0.01, ***p < 0.001; ns indicates no significant difference.

To investigate the molecular mechanisms by which the LTO/L/Ta interface promotes osteogenesis under immune modulation, whole‐transcriptome sequencing was performed on rBMSCs treated with macrophage‐derived conditioned media. Compared with the ‐Ta group, the +LTO/L/Ta group exhibited 3,943 significantly differentially expressed genes (DEGs), including 2,697 upregulated and 1,246 downregulated genes (Figure 7a). The volcano plot revealed a pronounced transcriptional shift toward genes associated with ECM remodeling, cell adhesion, and osteogenic differentiation, indicating extensive regenerative reprogramming under the immunomodulated microenvironment. Hierarchical clustering analysis of the DEGs further demonstrated that genes closely related to matrix adhesion, cytoskeletal organization, signal transduction, and osteogenic differentiation—such as *Itga1*, *Lama2*, *Col1a1*, *Itga4*, *Lamb1*, *Col1a2*, *Igf1*, *Vegfa*, *Pdgfb*, and *Ccnd1*—were markedly upregulated in the LTO/L/Ta and +LTO/L/Ta groups (Figure 7b). These results suggest that immune modulation induced by the piezoelectric LTO/L/Ta functionalized surface prominently activates ECM‐integrin‐mediated adhesion signaling, which may serve as a key upstream driver of osteogenic responses. Focal adhesion, believed to be the anchoring of cells on the ECM, is crucial for promoting cell adhesion through integrin binding in the ECM and simultaneously triggering intracellular pathways related to osteogenesis [46, 47]. KEGG pathway enrichment analysis further supported this notion, revealing significant activation of the PI3K–AKT signaling pathway, focal adhesion, ECM–receptor interaction, Rap1 signaling, and calcium signaling pathways (Figure 7c). These pathways are closely associated with mechanotransduction, adhesion strengthening, matrix assembly, and angiogenic regulation in osteoblasts, indicating that piezoelectric LTO/L/Ta functionalized surface, under immune modulation, orchestrate bone and vascular regeneration through coordinated, multi‐level signaling networks. Consistently, Gene Ontology (GO) enrichment analysis demonstrated significant enrichment of DEGs in biological processes related to cell–matrix adhesion, ECM organization, osteoblast differentiation, regulation of bone mineralization, initiation of angiogenesis, and responses to mechanical stimuli (Figure 7d). Cellular component analysis highlighted cytoskeleton, focal adhesions, ECM, and integrin complexes, while molecular function terms included growth factor binding, integrin binding, structural molecule activity, and receptor regulation. Together, these results indicate that immune‐mediated regeneration induced by LTO/L/Ta is likely associated with remodeling of the ECM–integrin–FAK/PI3K–Akt signaling axis, thereby enhancing cell adhesion, mechanosensitivity, and matrix deposition.

The remodeling of ECM not only plays an important role in the adhesion between cells and substrates, but also in cellular internalization and subsequent signal transduction processes [48]. ECM can act as a “zipper” between exogenous proteins and target cell integrins. Accordingly, to validate the transcriptomic findings, five representative ECM‐integrin‐related genes (*Itga1*, *Lama2*, *Col1a1*, *Itga4*, and *Lamb1*) were further examined by qPCR (Figure 7e). Compared with the control group, all five genes in the +LTO/L/Ta group were significantly upregulated. The increased expression of *Itga1* and *Itga4* indicates strengthened integrin‐mediated cell–ECM adhesion, while the upregulation of *Lama2* and *Lamb1* suggests reinforced basement membrane formation. Notably, the elevation of *Col1a1* directly confirms enhanced osteogenic ECM deposition, consistent with activation of upstream adhesion‐related signaling. At the protein level, immunofluorescence analysis further corroborated these molecular events. Compared with the control group, the +LTO/L/Ta group exhibited markedly increased COL1A1 deposition within the ECM, accompanied by more organized F‐actin cytoskeletal alignment (Figure 7f; quantified in Figure S78a). Collagen I (Col1) is a prominent ECM component that drives the formation of beaded microfibrillar networks within the ECM [49]. In parallel, integrin α1 (ITGA1) and the focal adhesion protein Vinculin showed significantly enhanced expression and more pronounced localization at cell–matrix contact sites (Figure 7g,h; quantified in Figure S78b,c), indicating promoted maturation and stabilization of integrin–focal adhesion complexes under immune modulation. Collectively, these results demonstrate that ultrasound‐activated piezoelectric LTO/L/Ta functionalized surface reshape macrophage paracrine signaling to activate the Collagen I–Integrin α1–Vinculin–FAK axis (Figure 7i), potentially facilitating rBMSCs adhesion and cytoskeletal reorganization. This integrin‐centered mechanoadhesive regulation provides a critical cellular foundation for subsequent osteogenic differentiation and bone regeneration. The proposed mechanism represents an integrated working model based on the collective evidence obtained from cellular and animal studies. The in vitro results elucidate the underlying cellular and molecular responses, whereas the in vivo findings validate the functional consequence of these coordinated biological processes during peri‐implant regeneration.

### 2.7 Angiogenesis Ability and Its Molecular Mechanism

**FIGURE 8:**
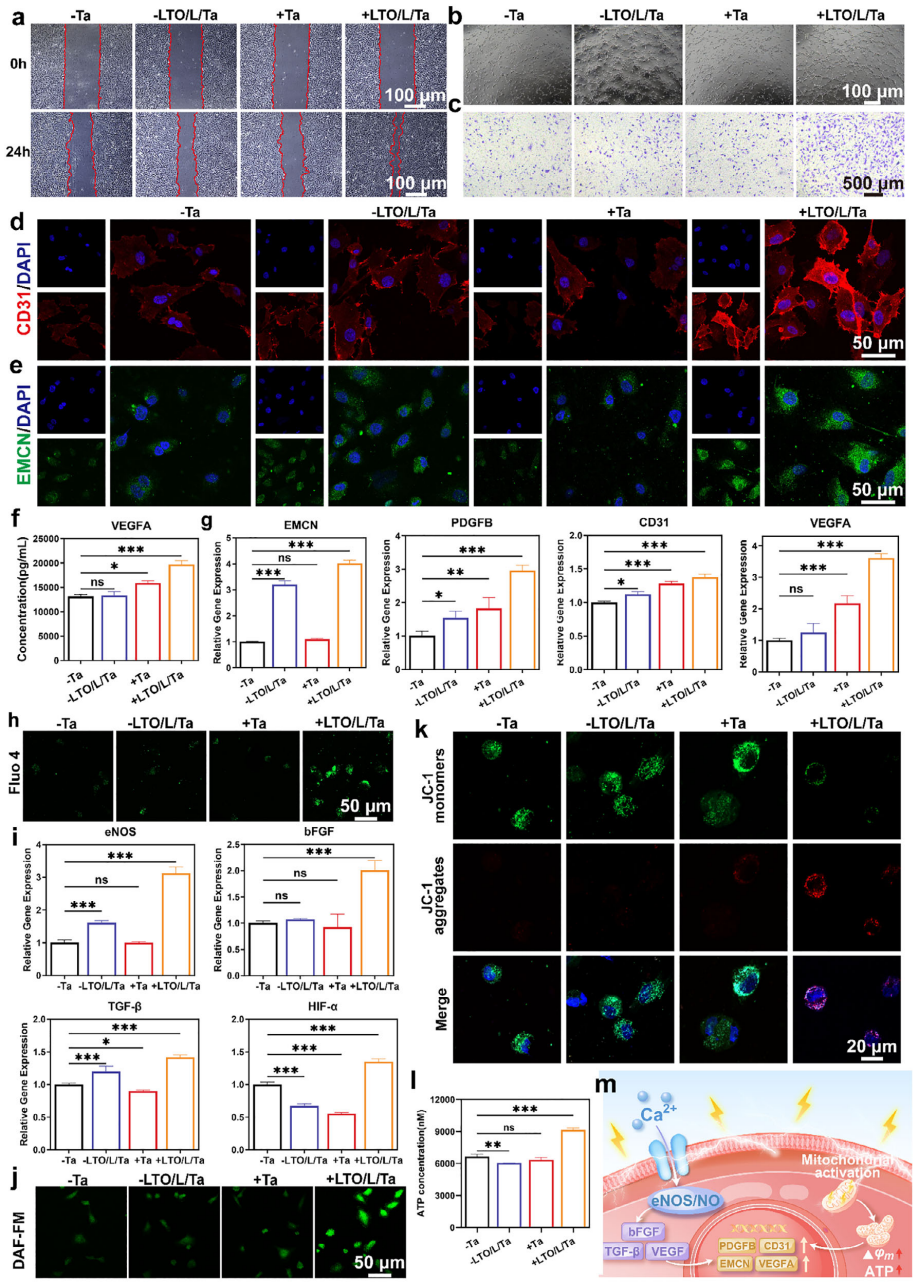
In vitro evaluation of angiogenic behaviors of endothelial cells induced by the force‐electric responsive LTO/L/Ta implant under LIPUS, together with elucidation of the underlying cellular and molecular mechanisms. a) Representative images of scratch wound healing assays at 0 and 24 h. b) Representative optical microscopy images of Matrigel tube formation assays. c) Transwell migration assay results showing endothelial cell migratory capacity. d) Immunofluorescence staining of CD31 expression in endothelial cells. e) Immunofluorescence staining of EMCN expression. f) Quantitative analysis of VEGFA secretion determined by ELISA. g) qRT‐PCR quantification of angiogenesis‐related genes, including *EMCN*, *PDGFB*, *CD31*, and *VEGFA*. h) Fluo‐4 calcium probe imaging showing intracellular Ca^2^^+^ fluorescence distribution in endothelial cells cultured on different material surfaces. i) qRT‐PCR analysis of angiogenic and signaling‐related genes (*bFGF*, *TGF‐β*, *HIF‐1α*, and *eNOS*). j) DAF‐FM DA staining for intracellular nitric oxide (NO) production. k) JC‐1 staining to assess mitochondrial membrane potential (ΔΨm), where green fluorescence indicates JC‐1 monomers and red fluorescence indicates JC‐1 aggregates. l) Quantification of intracellular ATP levels. m) Schematic illustration of the proposed mechanism by which LTO/L/Ta promotes angiogenesis under LIPUS. The data in (f), (g), (i), and (l) are presented as mean ± SEM, *n* = 3. *p*‐values are calculated using one‐way ANOVA followed by Tukey's post hoc test, *p < 0.05, **p < 0.01, ***p < 0.001; ns indicates no significant difference.

Angiogenesis is an indispensable prerequisite for successful bone regeneration, as endothelial activation and vascular network formation govern oxygen delivery, nutrient transport, and paracrine signaling within the regenerating microenvironment [50]. To investigate whether piezoelectric activation of the LTO/L/Ta interface could promote angiogenesis, the angiogenic functions of human umbilical vein endothelial cells (HUVECs) cultured on different surfaces were systematically evaluated under LIPUS stimulation. The LTO/L/Ta interface under LIPUS began with assessing endothelial cell viability using Live/Dead staining and CCK‐8 assays (Figure S79). All groups exhibited high cell viability; however, the LTO/L/Ta group and its ultrasonically stimulated counterpart (+LTO/L/Ta) showed higher cell density and more uniform cell spreading. Consistently, CCK‐8 assays (Figure S80) demonstrated that LTO/L/Ta significantly enhanced endothelial cell proliferation at days 3 and 5, with +LTO/L/Ta exhibiting the most pronounced effect, indicating early endothelial activation and proliferative response at the piezoelectric surface under LIPUS. The pro‐migratory effects of the surface were further evaluated using multiple migration assays. Scratch assays revealed that both LTO/L/Ta and +LTO/L/Ta markedly accelerated wound closure within 24 h compared with the control groups (Figure 8a), which was confirmed by quantitative analysis (Figure S81). In parallel, Matrigel tube formation assays (Figure 8b and Figure S82), together with quantitative tube length analysis (Figure S83), demonstrated enhanced capillary‐like network formation in the +LTO/L/Ta group. Transwell migration assays yielded consistent results, further confirming the strong pro‐migratory and pro‐angiogenic effects of the piezoelectric surface under LIPUS (Figure 8c). Endothelial activation and functional maturation were further validated by immunofluorescence staining. The fluorescence intensities of CD31, a marker of endothelial cell–cell junctions, and EMCN, a marker associated with endothelial glycocalyx and angiogenic endothelium, were significantly increased in the LTO/L/Ta group, with the strongest signals observed in the +LTO/L/Ta group (Figure 8d,e). Quantitative analysis (Figure S84) confirmed enhanced endothelial structural integrity and functional maturation. In addition, ELISA analysis showed that VEGFA secretion was significantly increased in the +LTO/L/Ta group (Figure 8f), indicating piezoelectric‐driven enhancement of pro‐angiogenic factor release. Gene expression analysis provided further mechanistic insight into the angiogenic response. qRT‐PCR results showed that key angiogenesis‐related genes, including *EMCN*, *PDGFB*, *CD31*, and *VEGFA*, were significantly upregulated on LTO/L/Ta, with the highest expression levels observed in the +LTO/L/Ta group (Figure 8g). These findings suggest that the piezoelectric functional surface promotes endothelial adhesion, migration, and growth factor expression in a coordinated manner. Notably, the laser‐oxidized Ta_2_O_5_ intermediate layer alone also induced moderate upregulation of angiogenic genes (Figure S85), indicating its contributory role in establishing a bioactive piezoelectric functional surface.

Intracellular calcium Ca^2+^ serves as a pivotal second messenger in HUVECs, orchestrating a variety of angiogenesis‐related processes [51]. Fluctuations in Ca^2+^ have been reported to regulate the activity of endothelial nitric oxide synthase (eNOS) and phospholipases, thereby modulating the secretion of vasoactive factors [52]. Moreover, accumulating evidence suggests that exogenous electrical stimulation‐induced endothelial responses, including cytoskeletal reorganization and directional migration, are also highly dependent on Ca^2+^ signaling. Given the bioelectrical cues generated by the piezoelectric LTO/L/Ta interface under LIPUS stimulation, we further explored whether Ca^2+^‐mediated signaling was involved in the enhanced angiogenic responses. To further elucidate the underlying mechanism, intracellular Ca^2^^+^ dynamics were examined using Fluo‐4 staining. Cells cultured on LTO/L/Ta exhibited stronger Ca^2^^+^ signals, which were further amplified under LIPUS(+LTO/L/Ta) (Figure 8h), as confirmed by quantitative analysis (Figure S86). Correspondingly, the expression of downstream Ca^2^^+^‐dependent angiogenic genes, including eNOS, bFGF, TGF‐β, and HIF‐1α, was significantly upregulated in the +LTO/L/Ta group (Figure 8i), indicating effective activation of calcium‐mediated signaling cascades. Given the central role of Ca^2^^+^ in eNOS/NO signaling, intracellular NO production was subsequently assessed by DAF‐FM staining. The +LTO/L/Ta group exhibited the strongest NO fluorescence intensity (Figure 8j), which was further confirmed by quantitative analysis (Figure S87), suggesting that piezoelectric stimulation promotes sustained NO generation through the Ca^2^^+^–eNOS axis to support angiogenic processes, aligning with the literature results [53]. To exclude the possibility that increased NO production was simply associated with variations in cell number, HUVECs were cultured under identical seeding density and experimental conditions. Cell viability and proliferation analyses showed no significant differences among groups (Figures S79 and S80). Therefore, the enhanced NO production observed in the +LTO/L/Ta group reflects improved endothelial functional activation rather than increased cell abundance. In parallel, mitochondrial activity was evaluated to assess the bioenergetic contribution to angiogenesis. JC‐1 staining revealed enhanced red fluorescence and reduced green fluorescence in the +LTO/L/Ta group, indicating elevated mitochondrial membrane potential (ΔΨm) (Figure 8k), consistent with the quantitative results (Figure S88). This was consistent with significantly increased intracellular ATP levels (Figure 8l), suggesting that piezoelectric‐induced mitochondrial activation provides sufficient energetic support for endothelial migration, tube formation, and angiogenic maturation. ATP production reflects the metabolic adaptation required for vascular activation and angiogenic progression. Endothelial migration, cytoskeletal remodeling, and tube formation are highly energy‐dependent processes that require continuous mitochondrial support. The elevated ATP levels observed in HUVECs after activation of the LTO/L/Ta interface may therefore contribute to enhanced endothelial functionality and angiogenic responses, together with the increased NO production and vascular‐related behaviors. Taken together, these results demonstrate that under LIPUS, LTO/L/Ta piezoelectric surface induce Ca^2^^+^ influx, synergistically activate the eNOS/NO signaling axis, and enhance mitochondrial energy metabolism, thereby coordinately upregulating multiple pro‐angiogenic factors. This integrated response establishes a “piezoelectric–Ca^2^^+^–NO–mitochondria–ATP” regulatory network that effectively drives endothelial angiogenesis (Figure 8m).

## 3 Conclusion

This work first manufactures an ultrasound‐activated Ta implant with force‐electric LiTaO_3_ nanotentacles by laser nanofabrication and in situ reaction, and reveals orchestrates vascularization bone regeneration of multi‐level molecular mechanism. Under controllable LIPUS stimulation, the force‐electric nanotentacles convert mechanical stimulation into localized bioelectric signals and provides nano‐anchoring sites. In BMSCs, this stimulation induces Ca^2^^+^ influx, activating PI3K–AKT signaling pathway and increasing mitochondrial ATP production to promote osteogenic differentiation. Meanwhile, integrin‐mediated PI3K–AKT activation drives macrophage polarization toward the M2 phenotype, establishing an actively pro‐regenerative osteoimmunology modulation microenvironment, while macrophage‐derived paracrine signaling further enhances osteogenesis through the ECM–integrin–FAK axis. In HUVECs, Ca^2^^+^‐dependent eNOS/NO signaling is enhanced, while increased mitochondrial ATP production promotes angiogenesis. Through coordinated regulation of macrophage polarization, osteogenic differentiation, and endothelial activation, the piezoelectric implant establishes a regenerative active microenvironment that promotes coupled angiogenesis and osteogenesis. Unlike previously reported piezoelectric implant systems that mainly focus on osteogenic differentiation, the force‐electric Ta implant enables real‐time, controllable multifunctional tissue regeneration through bioenergetic regulation and coordinated immune–vascular–bone crosstalk. By continuously converting physiological mechanical stimulation into localized bioelectric cues, this biomimetic strategy offers a promising platform for stable and long‐term osseointegration in patient‐personalized load‐bearing osteoarticular prostheses and dental implants. Further studies using larger animal models and clinically relevant implant configurations will be necessary to evaluate the long‐term safety, durability, and translational feasibility of this force‐electric implant system.

## 4 Experimental Section

### 4.1 Preparation of the Piezoelectric LTO/L/Ta Functionalized Surface on Ta

Ta was purchased from Qinghe Zhuan Metal Materials Co., Ltd. Anhydrous lithium hydroxide (AR, 98%) was obtained from Sinopharm Chemical Reagent Co., Ltd. All experiments used deionized water. High‐purity Ta sheets (99.95%, 1 mm thick) were cut into 10 mm × 10 mm samples and polished with 600–1500 grit sandpapers to remove surface oxides and damage. The samples were ultrasonically cleaned in acetone, ethanol, and deionized water for 30 min each, then dried with nitrogen. Laser surface direct writing was carried out using a 1064 nm laser with a 10 ns pulse width. The energy density was 15 J/cm^2^, the scanning speed was 0.8 mm/s, and the spot overlap was 50%. Processing was conducted under ambient conditions with a relative humidity of about 40%, forming a nanostructured Ta_2_O_5_ layer in situ. The treated samples were immersed in a Teflon‐lined autoclave containing 0.1 mol/L lithium hydroxide solution, filled to 80% volume. The autoclave was heated to 240 °C at 5 °C/min and maintained for 12 h. After natural cooling, the samples were washed three times with deionized water for 10 min each and vacuum‐dried at 60 °C for 2 h.

### 4.2 Magnetic Sputtering

The treated TC4 substrates and glass slides were placed in a vacuum chamber, which was evacuated to below 1 × 10^−^^3^ Pa. A circular Ta target (ϕ 100 mm × 5 mm, 99.95% purity) purchased from Zhongnuo New Materials (Beijing) was used. Argon plasma was applied for ion cleaning of both the samples and the target for 30 min. The argon pressure was maintained at 0.35 Pa, with a flow rate of 70 mL/min, and the distance from the sample center to the target center was 100 mm. During deposition, the samples rotated at 30 r/min to achieve a uniform Ta surface. A thermocouple attached to the back of a 2‐mm‐thick stainless steel holder monitored the deposition temperature. The substrate temperature was set at 180 °C, and a negative DC bias of 150 V was applied. Sputtering was performed with a current of 1 A and a power of 400 W. The interface thickness was controlled by deposition time, producing Ta films of 30 nm, 230 nm, and 1 µm.

### 4.3 Material Characterization Method

The surface and cross‐sectional morphologies were analyzed using a field‐emission scanning electron microscope (FESEM, Regulus8100). Interface was scraped onto copper grids and observed with a HRTEM (Talos F200i) to examine crystal morphology and lattice structure. Cross‐sectional samples were prepared using a focused ion beam (ZEISS Crossbeam 540) with a Ga^+^ beam ranging from 2.5 nA to 50 pA. Phase structures were identified by XRD (Smartlab SE) using Cu Kα radiation, a grazing incidence angle of 5°, and a scan range of 10°–90°. Chemical composition and valence states were measured by XPS (Kratos Axis Supra+) with an Al Kα source and Ar^+^ sputtering for depth profiling. Binding energies were calibrated against adventitious carbon (C 1s = 284.8 eV). Raman spectra were recorded on a Renishaw inVia system with a 532 nm laser at 1 mW, over a range of 100–1200 cm^−^^1^. Water contact angles were measured at room temperature by the sessile drop method using a contact angle goniometer (Dataphysics DCAT21), with at least five random points per sample. Piezoelectric properties were characterized by AFM (Bruker Dimension ICON) in PFM mode. The force‐electric response of the piezoelectric interface was quantified using a Keithley DMM 7510 digital multimeter, and transient voltage signals under ultrasound were recorded by a digital oscilloscope (UTD2202CEX+). Mechanical stability of the interface was evaluated by nanoindentation (Hysitron TI 950). Ultraviolet–visible–near‐infrared absorption spectra were collected using a Shimadzu UV‐3600 spectrophotometer.

### 4.4 FEM Simulations of the LiTaO_3_–Ultrasound Interaction

FEM analyses were carried out using COMSOL Multiphysics (V6.2), run on AMD 9B14 processor (2), with 384 GB RAM. The COMSOL “MEMS,” “Acoustics,” and “Structural Mechanics” modules were chosen to include the relevant physics of the acoustic pressure wave and the piezoelectric and dielectric response of the LiTaO_3_. Below is listed the detailed methods: All these modules presented a fully coupled steady‐state solution. Multiphysics environment of COMSOL and the steady‐state solutions (frequency domain solver) for electric field, stress, and strain were solved as a function of pressure. The radially poled configuration was used for LiTaO_3_‐Ta analyses. Linear piezoresponse and linear elastic media assumptions were adopted in the study. Piezoelectric strain‐charge formulation was used to solve for the piezoelectric response to stresses and strains induced by the acoustic field. The FEM model is shown in Figure 2q. Based on the experiment, LiTaO_3_ particles are attached to a tantalum substrate. The interaction between LiTaO_3_ particles and tantalum can be categorized into three scenarios: the first scenario involves a single LiTaO_3_ particle and tantalum; the second involves two LiTaO_3_ particles of identical size in contact with each other; and the third involves two LiTaO_3_ particles of different sizes. The corresponding overall model is divided into two layers. The upper layer consists of cubic LiTaO_3_ particles with sizes of 10 and 15 nm, subjected to a pressure of 140 kPa. The lower layer is a larger rectangular tantalum substrate. A fixed constraint is applied to the bottom surface, and the entire model is grounded.

### 4.5 Optimization of Ultrasound Parameters

Fourth‐generation Sprague–Dawley rBMSCs were seeded on sample surfaces in 24‐well plates at a density of 5 × 10^3^ cells/cm^2^. After 24 h of adhesion, the cells were treated with a 1 MHz ultrasound device according to experimental groups. Power gradients of 0.5, 1, 2, and 3 W/cm^2^ were applied for 5 min, and time gradients of 2, 5, and 10 min were applied at 3 W/cm^2^. Each group contained five replicates (*n* = 5). LIPUS stimulation was performed every two days. Cell viability was assessed using the CCK‐8 assay by measuring absorbance at 450 nm. Total RNA was extracted with the SteadyPure universal RNA kit (AG), reverse‐transcribed using the Evo M‐MLV kit (AG), and quantitative PCR was performed with the SYBR Green Pro Taq HS premix (AG) to measure relevant gene expression. Primer sequences are listed in Tables S1–S3. These results guided the selection of parameters for subsequent experiments.

### 4.6 Cell Culture

Primary rBMSCs were purchased from Wuhan Punosai Biotechnology Co., Ltd. and cultured in DMEM supplemented with 10% fetal bovine serum (FBS) and 1% penicillin–streptomycin. Passages 3–5 were used in experiments. RAW 264.7 cells were obtained from the same company and cultured in specialized medium for RAW 264.7. HUVECs were purchased from Shanghai Zhongqiao Xinzou Biotechnology Co., Ltd. and cultured in endothelial‐specific medium. HUVECs at passages 3–5 were used. All cells were incubated at 37 °C in a humidified atmosphere with 5% CO_2_.

### 4.7 Preparation of Conditioned Medium

Conditioned media were prepared by culturing RAW 264.7 cells for three days. RAW264.7 macrophages were seeded at an identical density and cultured under the indicated conditions for the same duration. Following stimulation, conditioned media were collected from each group using the same volume of culture medium, centrifuged to remove cellular debris, and subsequently used for downstream biological evaluation. The identical cell seeding density, culture duration, and collection conditions were maintained among all groups to minimize the influence of cell number variation. Media were collected from the experimental groups (+LTO/L/Ta), surface control groups (‐LTO/L/Ta), ultrasound control groups (+Ta), and blank control groups (‐Ta). These media were mixed 1:1 with DMEM or complete HUVEC medium to generate conditioned media for subsequent experiments.

### 4.8 Cell Proliferation and Morphological Characterization

Cell proliferation on the scaffolds was evaluated on days 1, 3, and 5 using a CCK‐8 kit (Biosharp). Absorbance at 450 nm was measured with a microplate reader to plot growth curves. After three days of culture, cells were stained with a live/dead assay (Calcein‐AM/PI, Yeasen) at room temperature for 15 min in the dark. Fluorescence was observed using an inverted microscope (SOPTOP, 10× objective). On day 3, the cell–scaffold complexes were fixed with 4% paraformaldehyde (PFA) and permeabilized with 0.1% Triton X‐100. The samples were then stained sequentially with FITC‐conjugated phalloidin (45 min, 37 °C) and DAPI (10 min, room temperature). The cytoskeleton morphology was reconstructed in three dimensions using a confocal laser scanning microscope (Zeiss CellDiscoverer 7). Parallel samples were fixed overnight at 4 °C in 4% PFA, dehydrated through an ethanol gradient (30%–100%), dried, and gold‐coated. The surface ultrastructure of the cells and filopodia extensions were observed using a FESEM at 5 kV.

### 4.9 Cell Energy Metabolism Detection

#### 4.9.1 Mitochondrial Membrane Potential Detection

Mitochondrial membrane potential of rBMSCs and HUVECs was assessed using a JC‐1 kit (Biyuntian). Cells were seeded on the sample surface in four groups: experimental (+LTO/L/Ta), surface control (‐LTO/L/Ta), ultrasound control (+Ta), and blank control (‐Ta). JC‐1 working solution was added according to the kit instructions. The cells were incubated at 37 °C with 5% CO_2_ for 20 min in the dark. After two washes with PBS, red and green fluorescence images were captured using a confocal laser scanning microscope. Fluorescence intensity ratios were quantified using ImageJ software.

#### 4.9.2 ATP Content Detection

ATP levels were measured in both rBMSCs and HUVECs. Cells were seeded in 96‐well plates at predetermined densities and co‐cultured with materials subjected to different surface treatments for specific durations. After incubation, the culture medium was discarded. According to the instructions of the Biyuntian ATP assay kit, 100 µL of lysis buffer was added to each well to lyse the cells. The supernatant was transferred to a new white 96‐well plate. Then, 20 µL of ATP detection working solution was added to each well and gently mixed with a pipette, avoiding bubbles. Chemiluminescence was measured immediately using a multifunctional microplate reader with a 1‐s integration time. Each experimental group included at least five replicates, and cell‐free lysis buffer served as a background control. ATP levels were expressed in relative luminescence units. After subtracting the background, values were normalized and analyzed relative to the control group.

### 4.10 Qualitative Detection of Osteogenic Differentiation

*ALP Staining*: rBMSCs were seeded at a density of 3 × 10^5^ cells/cm^2^ and induced for osteogenic differentiation under different treatment conditions, including direct sample stimulation and conditioned media. The induction medium contained 50 µg/mL ascorbic acid, 10 mm β‐glycerophosphate, and 100 nm dexamethasone, and cells were cultured for 7 days. After fixation with 4% PFA, ALP activity was visualized using a BCIP/NBT kit (Biyuntian) for 30 min in the dark. The coverage of blue‐purple precipitates was quantified under a microscope (*n* = 3). *ARS Staining*: After 21 days of osteogenic induction, cells were fixed with 4% PFA and stained using a calcium salt kit (Alizarin Red S, Solarbio) for 10–90 min. After washing with PBS, calcium nodules were imaged. The precipitates were then dissolved in 10% cetylpyridinium chloride solution, and absorbance at 562 nm was measured (*n* = 5). All samples included three replicates and four groups: experimental (+LTO/L/Ta), surface control (‐LTO/L/Ta), ultrasound control (+Ta), and blank control (‐Ta).

### 4.11 Immunofluorescence Staining

Immunofluorescence staining was performed to evaluate the expression of osteogenic proteins in rBMSCs, inflammatory markers in RAW 264.7 cells, and angiogenic proteins in HUVECs. Cells were fixed with 4% PFA at room temperature for 15 min and permeabilized with 0.2% Triton X‐100 for 15 min. To reduce nonspecific binding, cells were blocked with 5% goat serum at room temperature for 1 h. Primary antibodies against CD206 (M2 macrophage marker, Abcam), iNOS (M1 marker, Abcam), RUNX2 (osteogenic marker, Abcam), OPN (osteogenic protein, Abcam), CD31 (angiogenic marker, Wuhan Sanying), EMCN (angiogenic marker, Wuhan Sanying), COL1A1 (Ibotek), ITGA1 (Ibotek), and Vinculin (Wuhan Sanying) were added and incubated overnight at 4 °C. After washing with PBS, cells were incubated with 594 nm‐labeled secondary antibodies at 37 °C for 2 h in the dark. Nuclei were stained with DAPI (1 µg/mL) for 10 min, followed by three PBS washes to remove unbound dye. Samples were mounted with antifade reagent. Fluorescence images were acquired using a confocal laser scanning microscope (Zeiss CellDiscoverer 7). Multi‐channel fluorescence signals were overlaid and quantified using ZEN software and ImageJ.

### 4.12 Detection of Intracellular Calcium Ion (Ca^2^^+^) Concentration and Membrane Potential

After two days of sample treatment, cells were incubated with the calcium‐sensitive fluorescent probe Fluo‐4 AM and the membrane potential‐sensitive dye DISBAC_2_(3) (both from Beyotime) to label intracellular Ca^2^^+^ and monitor membrane potential changes. Probes were loaded at 37 °C for 30 min. Fluorescence images of the cells were then acquired using a confocal laser scanning microscope (Zeiss CellDiscoverer 7).

### 4.13 Enzyme‐Linked Immunosorbent Assay

After centrifugation of the culture medium, the supernatants were collected. Levels of IL‐10, TNF‐α, PDGF‐BB, BMP‐2, and VEGFA were measured using ELISA kits according to the manufacturers’ instructions. Supernatants from RAW 264.7 cells and HUVECs cultured on different samples were analyzed. The concentrations of secreted factors were determined by comparison with standard curves.

### 4.14 Transwell Invasion Experiment

HUVECs cultured for 5 days in direct stimulation or conditioned media from the experimental (+LTO/L/Ta), surface control (‐LTO/L/Ta), ultrasound control (‐Ta), and blank control (‐Ta) groups were collected and resuspended in serum‐free medium. Cell suspensions at a density of 2 × 10^4^ cells/mL were seeded into the upper chambers of Transwell inserts (8 µm pore size). The lower chambers (24‐well plate wells) contained complete HUVEC medium with 10% FBS. After 24 h incubation, nonmigrated cells on the upper membrane surface were carefully removed with a wet cotton swab. Cells that migrated to the lower membrane surface were fixed with 4% PFA for 20 min and stained with crystal violet (Beyotime). Migrated cells were observed and imaged under an optical microscope (CX 21, Olympus, Japan). Cell numbers were quantified using ImageJ software.

### 4.15 Scratch Assay

HUVECs cultured for 5 days in the experimental (+LTO/L/Ta), surface control (‐LTO/L/Ta), ultrasound control (+Ta), and blank control (‐Ta) groups were seeded in six‐well plates at a density of 5 × 10^5^ cells/mL. After reaching a confluent monolayer, a scratch was created by vertically dragging a 20 µL pipette tip across the cell layer. Images of the scratch area were captured immediately (0 h) and after 24 h using an optical microscope (CX 21, Olympus). The migration distance, representing the extent of wound closure, was quantified using ImageJ software.

### 4.16 In Vitro Microtubule Formation Experiment

Matrigel (BD Biosciences) was thawed at 4 °C overnight and evenly coated onto the wells of a 96‐well plate. The plate was incubated at 37 °C for 30 min to allow the Matrigel to fully polymerize. HUVECs cultured for 5 days under different treatment conditions, including direct sample stimulation and conditioned media, were collected and seeded onto the Matrigel‐coated wells at a density of 2 × 10^4^ cells per well. After 12 h of culture, tube formation was imaged using an optical microscope (CX 21, Olympus). Vascular network formation was quantified by measuring the number of branch points and the total capillary length using ImageJ software.

### 4.17 Nitric Oxide Detection

NO levels in HUVECs were measured using a DAF‐FM DA detection kit. HUVECs were seeded on the surfaces of different samples and cultured. DAF‐FM DA working solution was prepared according to the kit instructions and added to the cells, which were incubated at 37 °C for 30 min in the dark. Cells were washed three times with PBS to remove unincorporated probe. Fluorescence distribution was observed and imaged using a confocal laser scanning microscope. Fluorescence intensity was quantified using ImageJ to reflect relative NO levels.

### 4.18 Flow Cytometry

RAW 264.7 cells were treated with lipopolysaccharide (LPS) and exposed to different sample treatments for 48 h. After collection and centrifugation, cells were blocked with 3% bovine serum albumin (BSA) at room temperature for 20 min. Fluorescently labeled antibodies were then added for 20 min: APC‐conjugated anti‐CD86 (BioLegend) and PE‐conjugated anti‐CD206 (BioLegend). CD86 and CD206 were used as surface markers for M1 and M2 macrophages, respectively. After staining, cells were analyzed using a Cytek Aurora flow cytometer. Data were processed and analyzed with FlowJo V10.5.2 software.

### 4.19 Quantitative Real‐Time PCR

Total RNA was extracted from cells using SteadyPure Universal RNA Extraction Kit II (Accurate Biotechnology (Hunan) Co., Ltd., ChangSha, China, AG21022) according to the manufacturer's protocols. First‐strand cDNA was produced from 1 µg of total RNA from each sample using Evo M‐MLV RT Mix Kit with gDNA Clean for qPCR Ver.2 (Accurate Biotechnology (Hunan) Co., Ltd., ChangSha, China, AG1728). The mRNA levels of genes were analyzed using SYBR Green Premix Pro Taq HS qPCR Kit (Accurate Biotechnology (Hunan) Co., Ltd., AG11701) on Light Cycler@480 System (Roche, Switzerland).

### 4.20 Western Blot

rBMSCs cultured on different sample surfaces for 7 days were collected and lysed using RIPA buffer containing protease and phosphatase inhibitors (Solarbio). After centrifugation, the supernatant was collected as the protein sample. For each group, 30 µg of total protein was separated by SDS‐PAGE and transferred onto PVDF membranes (Merck Millipore). After blocking, membranes were incubated overnight at 4 °C with primary antibodies against RUNX2 (Abcam), OPN (Abcam), and GAPDH (Wuhan Sanying). The following day, membranes were washed and incubated with appropriate HRP‐conjugated secondary antibodies. Target protein bands were visualized using an ECL chemiluminescence kit (Millipore) on a chemiluminescence imaging system. Band intensities were quantified using ImageJ software.

### 4.21 RNA seq Analysis

rBMSCs cultured for 7 days on the experimental (+LTO/L/Ta) and blank control (‐Ta) surfaces, as well as in their respective conditioned media, were collected for total RNA extraction and transcriptome analysis. Total RNA was extracted using TRIzol Reagent (Invitrogen). Subsequent mRNA enrichment, fragmentation, reverse transcription, library construction, and sequencing on the HiSeq X Ten platform were performed by Guangzhou Chengqi Biotechnology Co., Ltd. Differentially expressed transcripts (DETs) were identified using a significance threshold of *P* < 0.05 and an absolute fold change ≥ 2 (|log_2_FC| ≥ 1). Functional annotation and pathway enrichment analyses of the DETs were performed. KEGG and GO enrichment analyses were conducted using the clusterProfiler R package. In addition, gene set enrichment analysis was performed using DESeq2 software to assess overall expression differences in predefined gene sets between the blank control (‐Ta) and experimental (+LTO/L/Ta) groups.

### 4.22 Animals

All animal procedures were approved by the Animal Ethics Committee of the Laboratory Animal Center, Shandong First Medical University (Ethics Approval No. L820250204), and were conducted in accordance with the Guide for the Care and Use of Laboratory Animals (China, 1996). Male Sprague–Dawley rats (10 weeks old, 290 ± 15 g) were used for the surgical experiments.

### 4.23 Experimental Study on Immune Regulation In Vivo

To evaluate the effects of different materials on the early inflammatory microenvironment, a rat subcutaneous implantation model was established. Male Sprague–Dawley rats were anesthetized via intraperitoneal injection of 2.0% avertin (300 mg/kg). The dorsal area was shaved and disinfected with povidone‐iodine. Two vertical incisions (1.5 cm each) were made symmetrically along the midline to create subcutaneous pockets. SLA‐treated titanium, pure Ta, and Ta with surface‐modified piezoelectric surface were implanted into the pockets. Incisions were sutured, and the day of surgery was designated as day 0. Starting from the day of surgery, the implantation sites were treated with ultrasound every other day (1 MHz, 1 W/cm^2^) for 10 min per session. All animals were euthanized, and samples were collected on postoperative day 7.

### 4.24 Experimental Study on Bone Integration and Vascularization around Screws In Vivo

Bilateral cylindrical defects (1 mm × 4.5 mm) were drilled into the femoral condyles of rats. To minimize variations in implant placement, all femoral defects were created at the same anatomical position using a standardized drilling procedure, and implants were inserted with consistent orientation and depth by the same operator. SLA‐treated titanium screws, pure Ta screws, and Ta screws with surface‐modified piezoelectric surface were implanted, one per femoral condyle, and the tissues were sutured layer by layer. The day of surgery was designated as day 0. Postoperatively, penicillin was administered intramuscularly for 3 days to prevent infection. Starting from postoperative day 7, the implantation sites were treated with ultrasound every other day (1 MHz, 1 W/cm^2^) for 5 min per session. Rats were euthanized under overdose anesthesia at 4 and 8 weeks after surgery.

### 4.25 X‐ray Analysis

Postoperative 4/8 weeks, femoral condyle X‐ray evaluation (Faxitron MX‐20) was performed, and positive/lateral images were collected.

### 4.26 Organizational Analysis

Subcutaneous tissue samples were collected and sectioned into consecutive transverse slices (5 µm thickness) for H&E and immunofluorescence staining. H&E staining was performed strictly according to the manufacturer's instructions (Servicebio, China). Immunofluorescence staining employed a homologous dual‐labeling method: paraffin sections were deparaffinized, and antigen retrieval was carried out using a one‐step deparaffinization/antigen repair buffer. Sections were then incubated sequentially with primary antibodies (anti‐iNOS, 1:200, Abcam; anti‐CD206, 1:300, Abcam), corresponding fluorescent secondary antibodies, and DAPI. Thorough washes were performed after each antibody incubation step.

Femoral samples from 4 and 8 week time points were fixed in 4% PFA for 48 h and processed in two ways. Undecalcified samples were dehydrated through a graded ethanol series, embedded in methyl methacrylate (MMA), and coronally sectioned at 5 µm. Decalcified samples were immersed in 10% EDTA solution (pH 7.4) for 4 weeks, with daily solution changes, and subsequently paraffin‐embedded.

### 4.27 Organizational Staining Processing

*Toluidine Blue–Basic Fuchsin*: Sections were stained with 0.1% toluidine blue (Sigma) at 37 °C for 30 min, followed by 0.01% basic fuchsin (Servicebio) for 10 min to assess bone matrix mineralization. H&E: Undecalcified sections were stained with hematoxylin (BH0001, Guoyao), blued with ammonia solution (1000211B, Guoyao), counterstained with eosin, dehydrated, and mounted. OPN immunofluorescence: Undecalcified sections were blocked with 3% BSA, incubated with rat anti‐OPN primary antibody (1:200, Abcam), followed by Alexa Fluor 488‐conjugated goat anti‐rat IgG secondary antibody (1:500), and DAPI nuclear counterstaining. Images were captured after mounting. Vascular markers in decalcified sections: CD31 immunohistochemistry involved incubation with rat anti‐CD31 (1:100, Abcam) and HRP‐conjugated goat anti‐rat secondary antibody (1:500), followed by DAB color development. EMCN immunofluorescence included overnight incubation at 4 °C with rat anti‐EMCN (1:200, Wuhan Sanying), followed by Alexa Fluor 488‐conjugated goat anti‐rat secondary antibody (1:500) in the dark and DAPI nuclear staining. Confocal laser scanning microscopy was used for image acquisition.

All sections were imaged using a Nikon Eclipse Ni‐E microscope. Immunopositive and immunofluorescent areas were quantified using ImageJ software. Bone morphometric analyses of Toluidine Blue–Basic Fuchsin‐stained sections were performed using the OsteoMeasure system.

### 4.28 RNA‐seq Data

The data generated in this study have been deposited in the Sequence Read Archive (SRA) of the National Center for Biotechnology Information (NCBI) with the accession number PRJNA1456784 and PRJNA1456899. The datasets will be publicly accessible upon publication of this article.

### 4.29 Artificial Intelligence Declaration

No artificial intelligence tools were used in the creation, generation, editing, or refinement of the figures or Table of Contents (ToC) image in this manuscript. All schematic diagrams and the ToC image were originally created by the authors using Adobe Illustrator 2025 and Cinema 4D. Cinema 4D was used for 3D modeling and rendering, while Adobe Illustrator 2025 was used for graphical composition, annotation, and refinement. Both software programs were used under valid and authorized licenses. No third‐party copyrighted graphical materials were incorporated into the figures or ToC image without appropriate permission or licensing. The authors take full responsibility for the originality and scientific accuracy of all graphical materials presented in this manuscript.

### 4.30 Statistical Analysis

All quantitative data were obtained from at least three independent biological replicates unless otherwise stated in the corresponding figure legends. Data are presented as mean ± SEM. No data transformation or normalization was performed for statistical analysis unless specifically indicated. Data distributions and experimental variations were carefully examined before statistical analysis, and no outliers were excluded from the datasets. Statistical analyses were performed using GraphPad Prism software (version 10.1.2). For comparisons between two groups, two‐tailed unpaired Student's *t*‐test was applied. For comparisons among multiple groups, one‐way analysis of variance (ANOVA) followed by Tukey's post hoc test was performed. All statistical tests were two‐sided, and a significance level of α = 0.05 was applied. Statistical significance was defined as *p < 0.05, **p < 0.01, ***p < 0.001, while “ns” indicates no significant difference. The exact sample size (*n*) and statistical methods used for each analysis are provided in the corresponding figure legends.

## Author Contributions

**Taixing Zhang**: writing – original draft, validation, investigation, data curation, conceptualization, visualization. **Ke Ma**: writing – original draft, methodology. **Kangqing Zuo**: writing – original draft, validation, funding acquisition. **Pandong Lin**: methodology, software, formal analysis. **Tailong Zhang**: writing – original draft, validation. **Rongliang Ding**: data curation. **Linbo Zhang**: validation. **Aonan Li**: resources, funding acquisition. **Yinchuan Wang**: methodology, funding acquisition. **Yanling Huang**: writing – original draft, validation. **Jichao Feng**: data curation, visualization. **Guiyong Xiao**: methodology. **Yupeng Lu**: writing – review and editing, investigation, funding acquisition. **Bing Han**: writing – review and editing, supervision, funding acquisition. **Zhiqiang Wang**: supervision, project administration. **Ningbo Li**: writing – review and editing, conceptualization, methodology, investigation, visualization, funding acquisition.

## Conflicts of Interest

The authors declare no conflicts of interest.

## Supporting information




**Supporting File**: advs77514‐sup‐0001‐SuppMat.docx.

## Data Availability

The data that support the findings of this study are available on request from the corresponding author. The data are not publicly available due to privacy or ethical restrictions.
