# Supplementary material for: Force‐Electric Nanotentacles of Tantalum Implant Accelerate Cellular Energy Metabolism/Crosstalk for Immunomodulation‐Vascularized Bone Integration
Source: Adv Sci (Weinh). 2026 Aug 30:e77514. Online ahead of print. doi: 10.1002/advs.77514 (PMC13526697; doi:10.1002/advs.77514)
Supplement: Supplementary file 1 [file ADVS-9999-e77514-s001.docx]

*Supporting Information*

**Force-Electric Nanotentacles of Tantalum Implant Accelerate Cellular Energy Metabolism/Crosstalk for Immunomodulation-Vascularized Bone Integration** Taixing Zhang^1,2,4,#^, Ke Ma^1,2,^^4,#^, Kangqing Zuo^1,2,4,#^, Pandong Lin^6,7^, Tailong Zhang^1,2,3^, Rongliang Ding^1,2,4^, Linbo Zhang^1,2,4^, Aonan Li^1,2,4^, Yinchuan Wang^8^, Yanling Huang^1,2,4^, Jichao Feng^1,2,4^, Guiyong Xiao^6,7^, Yupeng Lu^6,7^, Bing Han^2,5,^*, Zhiqiang Wang^1,2,3,^*, Ningbo Li^1,2,4,^*

^1^ Department of Stomatology, Shandong Provincial Hospital Affiliated to Shandong First Medical University, Ji’nan, Shandong Province 250021, P. R. China

^2^ School of Stomatology, Shandong First Medical University & Shandong Academy of Medical Sciences, Ji’nan, Shandong Province 250117, P. R. China

^3^ Department of Orthodontics, Shandong Provincial Hospital Affiliated to Shandong First Medical University, Ji’nan, Shandong Province 250021, P. R. China

^4^ Medical Science and Technology Innovation Center, Shandong First Medical University & Shandong Academy of Medical Sciences, Ji’nan, Shandong Province 250117, P. R. China

^5^ Department of Orthodontics, Cranial-Facial Growth and Development Center, Peking University School and Hospital of Stomatology & National Center of Stomatology & National Clinical Research Center for Oral Diseases & National Engineering Laboratory for Digital and Material Technology of Stomatology & Beijing Key Laboratory for Digital Stomatology & Research Center of Engineering and Technology for Computerized Dentistry Ministry of Health & NMPA Key Laboratory for Dental Materials, 22 Zhongguancun South Avenue, Haidian District, Beijing 100081, P.R. China

^6^ Key Laboratory for Liquid-Solid Structural Evolution and Processing of Materials, Ministry of Education, Shandong University, Ji’nan 250061, P. R. China

^7^ School of Materials Science and Engineering, Shandong University, Ji’nan 250061, P. R. China

^8^ School of Radiology, Shandong First Medical University & Shandong Academy of Medical Sciences, Taian, Shandong Province 250117, P. R. China

* Corresponding author.

Email addresses: kqbinghan@bjmu.edu.cn; wangzhiqiang@sdfmu.edu.cn; liningbo@sdfmu.edu.cn.

^#^ These authors contributed equally.

Figure S1. SEM images of surface morphology of tantalum substrate treated with different laser powers (25%, 30%, 35%, and 40%).

Figure S2. Cell viability of surfaces treated with different laser powers (CCK-8 method) was evaluated on days 1, 3, and 5 under conditions of ultrasound-free (-) or ultrasound-applied (+) (*p < 0.05, **p < 0.01, ***p < 0.001; ns denotes no significant difference).

Figure S3. XRD pattern of the intermediate oxide layer (L/Ta).

Figure S4. XPS spectrum of the intermediate oxide layer (L/Ta).

Figure S5. SEM images of surface morphology of lithium tantalate synthesized by hydrothermal synthesis on tantalum substrate surface at different reaction temperatures and reaction times.

Figure S6. Optical image of tantalum substrate surface treated by laser (L/Ta).

Figure S7. EDS elemental mapping (O and Ta) of the cross-section (prepared by FIB) of the layered structure.

Figure S8. Quantitative analysis of average surface roughness (Ra) of Ta and LTO/L/Ta (* * * p<0.001).

Figure S9. Raman spectrum of the LTO/L/Ta structure, showing characteristic Raman shifts at 207, 282, 314, 356, 380, 465, 596, 661, and 862 cm⁻¹.

Figure S10. High-resolution XPS spectra of LTO/L/Ta: a) O 1s spectrum (deconvoluted into peaks at 529.9 eV, 531.2 eV, and 532.6 eV); b) Ta 4f spectrum (deconvoluted into peaks at 25.7 eV and 27.6 eV).

Figure S11. SEM image of LTO/Ta sample (tantalum pretreated by air oxidation drying). b) EDS mapping of LTO/Ta.

Figure S12. XPS spectra of LTO/Ta (prepared via hydrothermal reaction after air oxidation-drying of Ta, part of universality verification).

Figure S13. Piezoelectric output signal curve of LTO/Ta (prepared via hydrothermal reaction after air oxidation-drying of Ta, part of universality verification).

Figure S14. Cross sectional SEM images and Si/Ta element EDS surface scan images of tantalum layers with different thicknesses (30nm,230nm,1μm) deposited by magnetron sputtering on silicon wafers.

Figure S15. Magnetron sputtering 30 nm tantalum layer: a) SEM image of Ta/Ti. b) EDS mapping of Ta/Ti. c) SEM image of L/Ta/Ti. d) EDS mapping of L/Ta/Ti. e) SEM image of LTO/Ta/Ti. f) EDS mapping of LTO/L/Ta/Ti.

Figure S16. XPS spectra of LTO/L/Ta/Ti samples coated with 30nm thick tantalum by magnetron sputtering spraying.

Figure S17. Atomic Force Microscopy (AFM) height imaging of the surface of LTO/L/Ta/Ti samples coated with a 30 nm-thick tantalum layer using magnetron sputtering coating technology. a) image is a three-dimensional height topography of the material surface. b) image is the corresponding two-dimensional height imaging map.

Figure S18. Piezoelectric output signal curve of LTO/L/Ta/Ti samples coated with 30nm thick tantalum by magnetron sputtering spraying.

Figure S19. Magnetron sputtering 230 nm tantalum layer: a) SEM image of Ta/Ti. b) EDS mapping of Ta/Ti. c) SEM image of L/Ta/Ti. d) EDS mapping of L/Ta/Ti. e) SEM image of LTO/L/Ta/Ti. f) EDS mapping of LTO/L/Ta/Ti.

Figure S20. XPS spectra of LTO/L/Ta/Ti samples coated with 230nm thick tantalum by magnetron sputtering spraying.

Figure S21. Atomic Force Microscopy (AFM) height imaging of the surface of LTO/L/Ta/Ti samples coated with a 230 nm-thick tantalum layer using magnetron sputtering coating technology. a) image is a three-dimensional height topography of the material surface. b) image is the corresponding two-dimensional height imaging map.

Figure S22. Piezoelectric output signal curve of LTO/L/Ta/Ti samples coated with 230nm thick tantalum by magnetron sputtering spraying.

Figure S23. XPS spectra of LTO/L/Ta/Ti samples coated with 1μm thick tantalum by magnetron sputtering spraying.

Figure S24. Atomic Force Microscopy (AFM) height imaging of the surface of LTO/L/Ta/Ti samples coated with a 1 μm-thick tantalum layer using magnetron sputtering coating technology. a) image is a three-dimensional height topography of the material surface. b) image is the corresponding two-dimensional height imaging map.

Figure S25. Piezoelectric output signal curve of LTO/L/Ta/Ti samples coated with 1μm thick tantalum by magnetron sputtering spraying.

Figure S26. The potential distribution of a size-mismatched nanoparticles shows dipole polarization (-9.52 to +7.6μV), where a) b) c) represent simulated potentials of different cross-sections, respectively.

Figure S27. Characterization results of nano scratch tester experiment: a) The force time curves of LTO/L/Ta and L/Ta nano scratch experiments are shown in the upper and lower figures, respectively. b) microscopic morphology of the scratch area.

Figure S28. a) Cross-sectional SEM image of the material. b) FIB haracterization results of the material: the left panel is the FIB imaging of the region, and the right panel is the corresponding line-scan elemental distribution curve.

Figure S29. Photograph of low-intensity pulsed ultrasound (LIPUS) applied to the rat femoral condyle defect model following implant placement.

Figure S30. SEM image of sandblasted acid etched titanium nail surface (SLA/Ti).

Figure S31. Hard tissue sections stained with toluidine blue-fuchsin basic, collected 4 weeks post-implantation of samples (SLA/Ti, -Ta, -LTO/L/Ta, +Ta, +LTO/L/Ta).

Figure S32 Quantitative analysis of bone contact index (BCI) at 4 weeks post-implantation for SLA/Ti, -Ta, -LTO/L/Ta, +Ta, and +LTO/L/Ta groups (*p < 0.05, **p < 0.01, ***p < 0.001; ns denotes no significant difference).

Figure S33. Immunofluorescence staining of OPN in hard tissue sections, collected 4 weeks post-implantation of samples (SLA/Ti, -Ta, -LTO/L/Ta, +Ta, +LTO/L/Ta).

Figure S34. Semi-quantitative analysis of OPN fluorescence intensity in hard tissue sections 4 weeks post-implantation of samples (SLA/Ti, -Ta, -LTO/L/Ta, +Ta, +LTO/L/Ta) (*p < 0.05, **p < 0.01, ***p < 0.001; ns denotes no significant difference).

Figure S35. Hard tissue sections (collected 4 weeks post-implantation of samples including SLA/Ti, -Ta, -LTO/L/Ta, +Ta, +LTO/L/Ta) stained with OPN first, followed by hematoxylin-eosin (HE) restraining after washing.

Figure S36. CD31 immunohistochemical staining of bone tissue corresponding to 4-week samples (SLA/Ti, -Ta, -LTO/L/Ta, +Ta, +LTO/L/Ta).

Figure S37. Semi-quantitative analysis of the positive area ratio of CD31 immunohistochemical staining in bone tissue 4 weeks post-implantation of samples (SLA/Ti, -Ta, -LTO/L/Ta, +Ta, +LTO/L/Ta) (*p < 0.05, **p < 0.01, ***p < 0.001; ns denotes no significant difference).

Figure S38. Immunofluorescence staining of bone tissue samples corresponding to 4 weeks using EMCN (SLA/Ti, -Ta, -LTO/L/Ta, +Ta, +LTO/L/Ta).

Figure S39. Quantitative analysis of EMCN fluorescence intensity at 4 weeks post-implantation for SLA/Ti, -Ta, -LTO/L/Ta, +Ta, and +LTO/L/Ta groups (*p < 0.05, **p < 0.01, ***p < 0.001; ns denotes no significant difference).

Figure S40. (a) Quantification of CD206-positive area ratio based on immunohistochemical staining. (b) Quantification of iNOS fluorescence intensity based on immunofluorescence staining.

Figure S41. Perform H&E staining on the heart, liver, spleen, lungs, and kidneys of mice in the +LTO/L/Ta, +Ta, -LTO/L/Ta, -Ta, and Ti (SLA) groups.

Figure S42. Optical photograph of LTO/L/Ta screw removed after 8 weeks of implantation in rat femur.

Figure S43. SEM of LTO/L/Ta screws removed after 8 weeks of implantation in rat femur.

Figure S44. Piezoelectric output signal curve of LTO/L/Ta screws removed after 8 weeks

Figure S45. Photograph of low-intensity pulsed ultrasound (LIPUS) applied to the rat subcutaneous dorsum model following implant placement.

Figure S46. The cell survival rates of Ta, L/Ta, and LTO/L/Ta surfaces (CCK-8 method) were evaluated at different ultrasound power densities (0, 0.5, 1, 2, 3 W/cm²) on days 1, 3, and 5 (*p < 0.05, **p < 0.01, ***p < 0.001; ns denotes no significant difference).

Figure S47. qPCR analysis of relative expression levels of osteogenic related genes at 7 days, detecting Ta and LTO/L/Ta surfaces treated with different ultrasound power densities (*p < 0.05, **p < 0.01, ***p < 0.001; ns denotes no significant difference).

Figure S48. qPCR analysis of relative expression levels of osteogenic related genes at 7 days, detecting Ta and LTO/L/Ta surfaces treated with different ultrasound times (2, 5, 10 min) (*p < 0.05, **p < 0.01, ***p < 0.001; ns denotes no significant difference).

Figure S49. qPCR analysis of relative expression levels of osteogenic related genes at 14 days was performed on Ta and LTO/L/Ta surfaces treated with different ultrasound times (2, 5, 10 min) (*p < 0.05, **p < 0.01, ***p < 0.001; ns denotes no significant difference).

Figure S50. Cell viability (assessed by CCK-8 assay, OD 450 nm) of BMSCs cultured on Ta and LTO/L/Ta surfaces under ultrasound-free (-) or ultrasound-applied (+) conditions at 1-, 3-, and 5-days post-seeding (*p < 0.05, **p < 0.01, ***p < 0.001; ns denotes no significant difference).

Figure S51. Cell Live/Dead staining images of BMSCs cultured on Ta and LTO/L/Ta surfaces under ultrasound-free (-) or ultrasound-applied (+) conditions. Live cells are stained green.

Figure S52. Cytoskeleton staining images of BMSCs cultured on Ta and LTO/L/Ta surfaces under ultrasound-free (-) or ultrasound-applied (+) conditions. F-actin is stained red, and cell nuclei (stained with DAPI) are blue.

Figure S53. Semi-quantitative analysis of cell spreading area for BMSCs cultured on Ta and LTO/L/Ta surfaces under ultrasound-free (-) or ultrasound-applied (+) conditions (*p < 0.05, **p < 0.01, ***p < 0.001; ns denotes no significant difference).

Figure S54. SEM images of cell morphology on Ta and LTO/L/Ta surfaces under conditions of ultrasound-free (-) or ultrasound-applied (+).

Figure S55. ALP staining results of Ta and LTO/L/Ta surface BMSCs under ultrasound-free (-) or ultrasound-applied (+) conditions.

Figure S56. Alizarin red staining results of Ta and LTO/L/Ta surface BMSCs under ultrasound-free (-) or ultrasound-applied (+) conditions.

Figure S57. qPCR analysis of relative expression levels of osteogenic related genes at 14 days, detecting Ta and LTO/L/Ta surface under ultrasound-free (-) or ultrasound-applied (+) conditions (*p < 0.05, **p < 0.01, ***p < 0.001; ns denotes no significant difference).

Figure S58. Immunofluorescence staining of osteogenic markers in cells cultured on Ta and LTO/L/Ta surface under ultrasound-free (-) or ultrasound-applied (+) conditions: a) RUNX2 (red) and DAPI (blue); b) OPN (green) and DAPI (blue).

Figure S59. Quantitative analysis of osteogenic marker fluorescence intensity corresponding to immunofluorescence staining: a) Fluorescence intensity of RUNX2; b) Fluorescence intensity of OPN (*p < 0.05, **p < 0.01, ***p < 0.001; ns denotes no significant difference).

Figure S60. qPCR analysis of the relative expression levels of osteogenic related genes at 7 days with the detection targets being the original Ta surface and L/Ta surface (*p < 0.05, **p < 0.01, ***p < 0.001; ns denotes no significant difference).

Figure S61. qPCR analysis of the relative expression levels of osteogenic related genes at 14 days, with the detection targets being the original Ta surface and L/Ta surface (*p < 0.05, **p < 0.01, ***p < 0.001; ns denotes no significant difference).

Figure S62. Volcano plot of transcriptome sequencing results: the x-axis denotes log2 (Fold Change), and the y-axis denotes -log10 (P-value). Red dots (Sig_Up, 634) represent significantly upregulated genes, blue dots (Sig_Down, 744) represent significantly downregulated genes, and gray dots (NoDiff, 12885) represent genes with no significant difference.

Figure S63. Bar plot of GO functional enrichment analysis, covering biological process (BP), cellular component (CC), and molecular function (MF). The x-axis denotes the number of enriched genes (Counts) , the y-axis represents GO terms, and color intensity corresponds to -log10 (p-value). Key enriched terms are highlighted by red boxes.

Figure S64. a) Intracellular Ca²⁺ levels detected by the Fluo-4 fluorescent probe of BMSCs cultured on Ta and LTO/L/Ta surfaces under ultrasound-free (-) or ultrasound-applied (+) conditions. b) Cell membrane potential assessed using the DiBAC4(3) fluorescent probe of BMSCs cultured on Ta and LTO/L/Ta surfaces under ultrasound-free (-) or ultrasound-applied (+) conditions.

Figure S65. Under ultrasound-free (-) or ultrasound-applied (+) conditions, the intracellular calcium ion concentration (Ca²^+^) on the surface of Ta and LTO/L/Ta BMSCs (*p < 0.05, **p < 0.01, ***p < 0.001; ns denotes no significant difference).

Figure S66. Quantitative analysis of surface cell membrane potential of Ta and LTO/L/Ta under ultrasound-free (-) or ultrasound-applied (+) conditions (*p < 0.05, **p < 0.01, ***p < 0.001; ns denotes no significant difference).

Figure S67. qPCR analysis of relative expression levels of inflammation related genes was performed on Ta and LTO/L/Ta surfaces treated with different ultrasound times (2 min, 5 min, 10 min) (*p < 0.05, **p < 0.01, ***p < 0.001; ns denotes no significant difference).

Figure S68. CCK-8 analysis of RAW 264.7 viability at 1, 2, and 3 days.

Figure S69. Live and dead staining results of macrophages on Ta and LTO/L/Ta surfaces under ultrasound-free (-) or ultrasound-applied (+) conditions.

Figure S70. SEM morphology images of Ta and LTO/L/Ta surface macrophages under ultrasound-free (-) and ultrasound-free (+) conditions.

Figure S71. a) Quantitative analysis of CD206 fluorescence intensity. b) Quantitative analysis of iNOS fluorescence intensity.

Figure S72. qPCR analysis of relative expression levels of immune regulation related genes on the surface of primitive Ta and L/Ta (*p < 0.05, **p < 0.01, ***p < 0.001; ns denotes no significant difference).

Figure S73. Expression of angiogenesis-related genes (*EMCN*, *CD31*, *VEGFA*) in HUVECs at 7 days from angiogenesis crosstalk mediated by immunomodulation (*p < 0.05, **p < 0.01, ***p < 0.001; ns denotes no significant difference).

Figure S74. a) Visualization of HUVECs migration at 0 h and 24 h in immunomodulation-mediated angiogenic crosstalk. b) Wound closure rate of HUVECs in immunomodulation-mediated angiogenic crosstalk.

Figure S75. Staining images of endothelial tube formation assays from angiogenesis crosstalk mediated by immunomodulation.

Figure S76. Total tube length from angiogenesis crosstalk mediated by immunomodulation (*p < 0.05, **p < 0.01, ***p < 0.001; ns denotes no significant difference).

Figure S77. Transwell migration assay staining results from angiogenesis crosstalk mediated by immunomodulation.

Figure S78. Fluorescence intensity of markers in BMSC: a) Fluorescence intensity of Col1a1; b) Fluorescence intensity of Itga1; c) Fluorescence intensity of Vinculin (*p < 0.05, **p < 0.01, ***p < 0.001; ns denotes no significant difference).

Figure S79. Cell viability (assessed by CCK-8 assay, OD450 nm) of HUVECs cultured on Ta and LTO/L/Ta surfaces under ultrasound-free (-) or ultrasound-applied (+) conditions at 1, 3, and 5 days (*p < 0.05, **p < 0.01, ***p < 0.001; ns denotes no significant difference).

Figure S80. Cell Live/Dead staining images of HUVECs cultured on Ta and LTO/L/Ta surfaces under ultrasound-free (-) or ultrasound-applied (+) conditions. Live cells are stained green.

Figure 81. Wound closure rate of HUVECs cultured on Ta and LTO/L/Ta surfaces under ultrasound-free (-) or ultrasound-applied (+) conditions (assessed by scratch assay) (*p < 0.05, **p < 0.01, ***p < 0.001; ns denotes no significant difference).

Figure S82. Under the conditions of ultrasound-free (-) or ultrasound-applied (+), microscopic images of Ta and LTO/L/Ta surfaces in tube forming experiments.

Figure S83. Total tube length of HUVECs cultured on Ta and LTO/L/Ta surfaces under ultrasound-free (-) or ultrasound-applied (+) conditions (assessed by tube formation assay) (*p < 0.05, **p < 0.01, ***p < 0.001; ns denotes no significant difference).

Figure S84. Fluorescence intensity of endothelial markers in HUVECs cultured on Ta and LTO/L/Ta surfaces under ultrasound-free (-) or ultrasound-applied (+) conditions: (a) Fluorescence intensity of CD31; (b) Fluorescence intensity of EMCN (*p < 0.05, **p < 0.01, ***p < 0.001; ns denotes no significant difference).

Figure S85. qPCR analysis of relative expression levels of angiogenesis related genes on the original Ta surface and L/Ta surface (*p < 0.05, **p < 0.01, ***p < 0.001; ns denotes no significant difference).

Figure S86. Intracellular calcium ion ([Ca+]) fluorescence intensity of HUVECs cultured on Ta and LTO/L/Ta surfaces under ultrasound-free (-) or ultrasound-applied (+) conditions. *** denotes p < 0.001.

Figure S87. Fluorescence intensity of DAF-FM (used to evaluate intracellular nitric oxide (NO) level) in HUVECs cultured on Ta and LTO/L/Ta surfaces under ultrasound-free (-) or ultrasound-applied (+) conditions (*p < 0.05, **p < 0.01, ***p < 0.001; ns denotes no significant difference).

Figure S88. JC-1 monomers/aggregates fluorescence intensity ratio (used to evaluate mitochondrial membrane potential) of HUVECs cultured on Ta and LTO/L/Ta surfaces under ultrasound-free (-) or ultrasound-applied (+) conditions (*p < 0.05, **p < 0.01, ***p < 0.001; ns denotes no significant difference).

**Table S1** The sequences of primers related to osteogenic differentiation for qPCR.

|  | **Gene** | **Species** | **Sequence (5'→3') F** | **Sequence (5'→3') R** |
| --- | --- | --- | --- | --- |
|  | GAPDH | Rat | GGCACAGTCAAGGCTGAGAATG | ATGGTGGTGAAGACGCCAGTA |
|  | OPN | Rat | GCCGAGGTGATAGCTTGGCTTA | TTGATAGCCTCATCGGACTCCTG |
|  | Runx2 | Rat | CATGGCCGGGAATGATGAG | TGTGAAGACCGTTATGGTCAAAGTG |
|  | Col1a1 | Rat | GACATGTTCAGCTTTGTGGACCTC | AGGGACCCTTAGGCCATTGTGTA |
|  | Bmp2 | Rat | ACATCCACTCCACAAACGAGAAA | CCACATCACTGAAGTCCACATACA |
|  | Alpl | Rat | ACAACACCAACGCTCAGGTC | GTGACCTCGTTCCCCTGAGT |
| Itga1 | | Rat | TGAATGTCTCGCTCCTCCTG | ATATCTGAATAGCCAGCTCTCG |
| Itga4 | | Rat | CTCCTTGTTGGAGCGCCTAT | CCGACGAGCACTCTTTCCAT |
| Lama2 | | Rat | CTGGAAACAACTTTGGTCTTGACT | ACATTGACTTCTGGCCTTGCT |
| Lamb1 | | Rat | GTGGACAGGAGCAATGAGGA | GTTCGCAACGGCTTCAATAC |
| ItgaV | | Rat | CCAGACCTGTTGTCACCGTA | CCCTTTGCCATCCGCCTTTA |
| Itgb3 | | Rat | TGACGAAGTGAAGAAGCAGAGT | GCATCATTCCTCCAGCCAATT |
| Pik3r3 | | Rat | TCCACCAAAGCCACCTAAGC | ATTTGTCATTTACCTCTTCCCTGG |
| Akt1 | | Rat | ATGGACTTCCGGTCAGGTTCA | GCCCTTGCCCAGTAGCTTCA |
| Bcl2l1 | | Rat | CTGGTGGTTGACTTTCTCTCCTAC | GTCTCCCTTTCTGGTTCAGTTTCT |
|  | Pdgfra | Rat | CACTTGCTATTACAACCACACTCA | CAGAATCGTCCTCTTCCACAATG |

**Table S1** **(Continued)** The sequences of primers related to osteogenic differentiation for qPCR.

| **Gene** | **Species** | **Sequence (5'→3') F** | **Sequence (5'→3') R** |
| --- | --- | --- | --- |
| Pdgfb | Rat | CTCGATGGGCAGAGATTCACA | TCTCATAGCGTGGTTTCCTC |
| Fgfr2 | Rat | GACCACGAATGGGCGACT | CCCACATTAACACCCCGAAG |
| Spp1 | Rat | GCTGAAGCCTGACCCATCTC | TGGAGTTGCTTGGAAGAGTTTCT |
| Pik3ca | Rat | ACCTCAGGCTTGAAGAGTGTC | CAGATGTTCTCCATGATTCGGA |

**Table S2** The sequences of primers related to immune regulation for qPCR.

|  | **Gene** | **Species** | **Sequence (5'→3') F** | **Sequence (5'→3') R** |
| --- | --- | --- | --- | --- |
| GAPDH | | Mus | TGTGTCCGTCGTGGATCTGA | TTGCTGTTGAAGTCGCAGGAG |
| iNOS | | Mus | ACTACTGCTGGTGGTGACAA | GAAGGTGTGGTTGAGTTCTCTAAG |
| TNF-α | | Mus | ACTCCAGGCGGTGCCTATGT | GTGAGGGTCTGGGCCATAGAA |
| IL-1β | | Mus | AGACAACTGCACTACAGGCT | TTGTCGTTGCTTGGTTCTCCTT |
| Arg-1 | | Mus | AGCTCTGGGAATCTGCATGG | ATGTACACGATGTCTTTGGCAGATA |
| IL-10 | | Mus | GCCAGAGCCACATGCTCCTA | GATAAGGCTTGGCAACCCAAGTAA |
| CD206 | | Mus | ACGAGCAGGTGCAGTTTACA | ACATCCCATAAGCCACCTGC |
| IL-6 | | Mus | CCACTTCACAAGTCGGAGGCTTA | TGCAAGTGCATCATCGTTGTTC |
| Tgfb1 | | Mus | CCCGAAGCGGACTACTATGC | TGCTTCCCGAATGTCTGACG |
| Vegfa | | Mus | GGGATCTTGCATCGGACCAG | AGAGCCCAGAAGTTGGACGA |
| Bmp2 | | Mus | TTCCATCACGAAGAAGCCGT | GAAACTCGTCACTGGGGACA |
| Pdgfb | | Mus | GCTGCTGCAATAACCGCAA | CAATCTCAATCTTTCTCACCTGGAC |
| Cd31 | | Mus | TCCAACAGAGCCAGCAGTATG | CCAATGACAACCACCGCAATG |

**Table S3** The sequences of primers related to angiogenesis for qPCR.

| **Gene** | **Species** | **Sequence (5'→3') F** | **Sequence (5'→3') R** |
| --- | --- | --- | --- |
| GAPDH | Homo | GTGAAGGTCGGAGTCAACGG | GCAACAATATCCACTTTACCAGAGT |
| VEGFA | Homo | GGGCAGAATCATCACGAAGTGG | AAGATGTCCACCAGGGTCTCG |
| CD31 | Homo | ACGTGCAGTACACGGAAGTTCA | AGGGAGCCTTCCGTTCTAGAGT |
| PDGFB | Homo | TGGCATGCAAGTGTGAGACAG | CGAATGGTCACCCGAGTTTGG |
| NOS3 | Homo | GCTGTCTGCATGGACCTGGATA | ATGGTGACTTTGGCTAGCTGGT |
| bFGF | Homo | GTGTGCTAACCGTTACCTGGCTATG | CCAGTTCGTTTCAGTGCCACA |
| TGF-β | Homo | ATTCCTGGCGATACCTCAGC | CGGTAGTGAACCCGTTGAT |
| HIF-1α | Homo | CTCATCAGTTGCCACTTCCACATA | AGCAATTCATCTGTGCTTTCATGTC |
| EMCN | Homo | CTGGTGGGTTTGTACCGAATGT | GCTTCACGCTCTCTTTATCAGACT |
